# Filter cake extract from the beet sugar industry as an economic growth medium for the production of *Spirulina platensis* as a microbial cell factory for protein

**DOI:** 10.1186/s12934-023-02146-7

**Published:** 2023-07-24

**Authors:** Sara Saad, Mervat Hosny Hussien, Ghada Samir Abou-ElWafa, Heshmat Soliman Aldesuquy, Eladl Eltanahy

**Affiliations:** Botany Department, Faculty of Science, Mansoura University, Mansoura, 35516 Egypt

**Keywords:** Sugar beet, Beet filter cake, Single cell protein, *Spirulina platensis*

## Abstract

**Background:**

Beet filter cake (BFC) is a by-product of sugar beet processing, which is difficult to dispose of and involves severe environmental concerns. *Spirulina platensis* is a microalga with a high protein content essential for human and animal nutrition. The present study aimed to utilize the beet filter cake extract (BFCE) to produce *Spirulina platensis* commercially*.* However, the cultivation of *S. platensis* on BFCE to produce economically single-cell protein has not been reported previously.

**Results:**

The batch experiment revealed the maximum dry weight at Zarrouk’s medium (0.4 g/L) followed by 0.34 g/L in the treatment of 75% BFCE. The highest protein content was 50% in Zarrouk’s medium, followed by 46.5% in 25% BFCE. However, adding a higher concentration of 100% BFCE led to a protein content of 31.1%. In the adaption experiment, *S platensis* showed an increase in dry cell weight and protein content from 25 to 75% BFCE (0.69 g/L to 1.12 g/L and 47.0% to 52.54%, respectively) with an insignificant variation compared to Zarrouk’s medium (p ≤ 0.05), indicating that *S. platensis* can be economically produced when cultivated on 75% BFCE The predicated parameters from response surface methodology were NaNO_3_ (2.5 g/L), NaHCO_3_ (0.67 g/L), BFCE (33%) and pH = 8, which resulted in biomass yield and protein content (0.56 g/L and 52.5%, respectively) closer to that achieved using the standard Zarrouk’s medium (0.6 g/L and 55.11%). Moreover, the total essential amino acid content was slightly higher in the optimized medium (38.73%) than SZM (36.98%).

**Conclusions:**

Therefore, BFCE supplemented medium could be used as a novel low-cost alternative growth medium for producing a single-cell protein with acceptable quantity and quality compared to the standard Zarrouk’s medium.

**Graphical Abstract:**

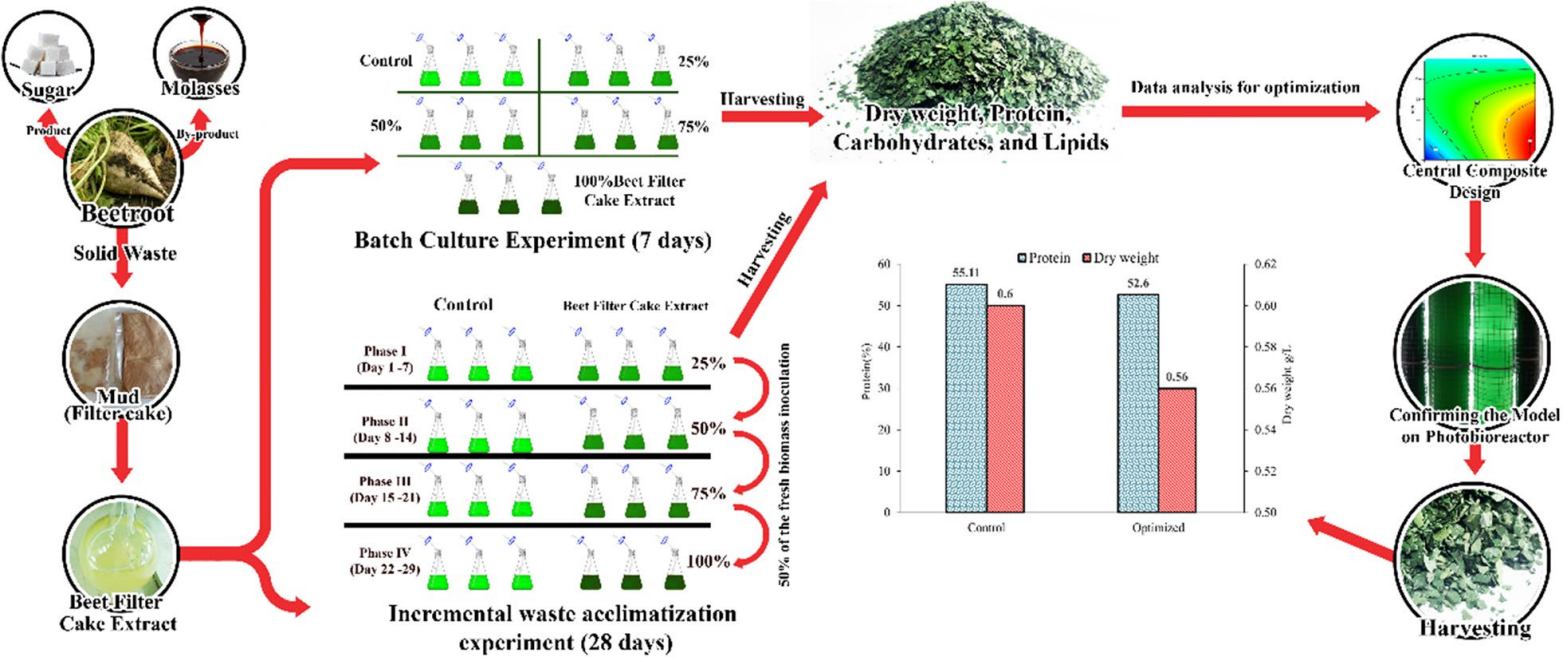

**Supplementary Information:**

The online version contains supplementary material available at 10.1186/s12934-023-02146-7.

## Background

Sugar cane and sugar beet account for most of the sugar produced in Egypt and worldwide. Sugar beet was grown on 207,527 hectares in 2019 and is Egypt's largest source of sugar production. The sugar industry starts with sucrose extraction from sugar beet using hot water resulting in raw juice, which is then purified, filtered, and concentrated by cyclic rinsing and evaporation. During the clarification of beet juice, milk of lime and sulfur dioxide are added to neutralize the juice's acidity and precipitate the non-sugar components. The residue is settled in a clarifier, and the settled sludge is filtered using a rotary vacuum filter [1].

Solid waste is produced as a by-product of the clarification of juice before its concentration and crystallization, called beet filter cake (BFC), also known as press mud, which is relatively rich in organic matter and some minerals causing a disposal problem [2]. The sugar factories dispose of these wastes in the open landscapes polluting the natural environment of that area. Therefore, recycling this waste is essential to reduce environmental pollution significantly and improve sustainability [1, 3]. Many scholars have studied the effect of BFC on biodiesel [4], crop productivity, soil fertility, and the possibility of its utilization as an organic fertilizer, but it is limited due to transport costs and soil acidity [5, 6].

*Spirulina platensis* is an economically crucial filamentous cyanobacterium naturally grown in alkaline water and warm regions and commercially produced for human and animal consumption [7, 8]. The *Spirulina* deserves special attention as a microbial cell factory for edible protein production (70% of dry biomass), essential amino acids, fats, and carbohydrates [9]. Moreover, bioactive compounds such as phycocyanin, chlorophylls and phenolic compounds benefit human health [10, 11] *S. platensis* commercial cultivation on the standard Zarrouk’s medium as a selective medium that minimizes contamination requires excessive nutrients, especially NaHCO_3,_ for both carbon source and pH control. The production cost of the growth medium is relatively high because NaHCO_3_ concentration is 16.8 kg/m^3^, which is a limiting factor for large-scale cultivation and economic feasibility [12].

Single-cell protein (SCP) is a dried biomass of high protein content that can be used as an alternative protein in animal feed or human nutrient supplements. Different microorganisms like yeast, bacteria and cyanobacteria can produce SCP, such as *S. platensis* [13]. The commercial trend for SCP production depends on using wastes from agriculture and food residues, which are cheap, non-toxic, renewable substrates, resulting in biomass of high protein content and waste management [14]. Utilizing the BFC in cultivating *S. platensis* as a microbial cell factory for single-cell protein has not been reported in the literature. Therefore, this study aims to formulate a new cost-effective medium using BFC supplementation which can minimize environmental pollution and production costs as possible while maintaining high biomass and protein content by optimization of the different growth variables incorporated in the medium for maximum biomass and protein yield in both photobioreactors and outdoor open ponds.

## Results and discussion

### BFCE analysis

Analyzing the BFCE components is critical because detecting toxic heavy elements or other pollutants will result in harmful, unaccepted *S. platensis* alga products in food or feed applications. Since this waste is from one of the clean food industries' wastes, the result of the analysis of the various elements in the extract using inductively coupled plasma mass spectrometry (ICP-MS) was as expected and at a minimum level; moreover, mercury, cadmium, and copper were not detected. On the other hand, the Ca^++^ and Na^+^ were at 565 ppm and 391 ppm, respectively, which are acceptable values. Also, ammonia, nitrate, and phosphate were 2.05 ppm, 4.84 ppm and 50.1 ppm, respectively (Table 1).

**Table 1:** Inductively coupled plasma mass spectrometry analysis of elements NH_3_, NO_3_, and P in BFCE

Element	Concentration (ppm)	Element	Concentration (ppm)
Al	271.9	Fe	22.848
Se	0.258	Ga	0.51
V	0.482	In	5.766
Bi	0.916	K	34.874
Hg	ND	Li	0.275
Ag	0.749	Mg	55.975
B	0.97	Mn	0.987
Ba	0.285	Ni	2.816
**Ca**	565.7	Pb	2.3
Cd	ND	Sr	0.879
Co	0.149	Zn	1.414
Cr	13.614	As	0.194
Cu	ND	Na	391.06
NH_3_	2.05	NO_3_	4.84
P	50.1		

*ND *none detected elements

### Batch culture experiment

#### Photosynthetic activity

**Fig. 1:**
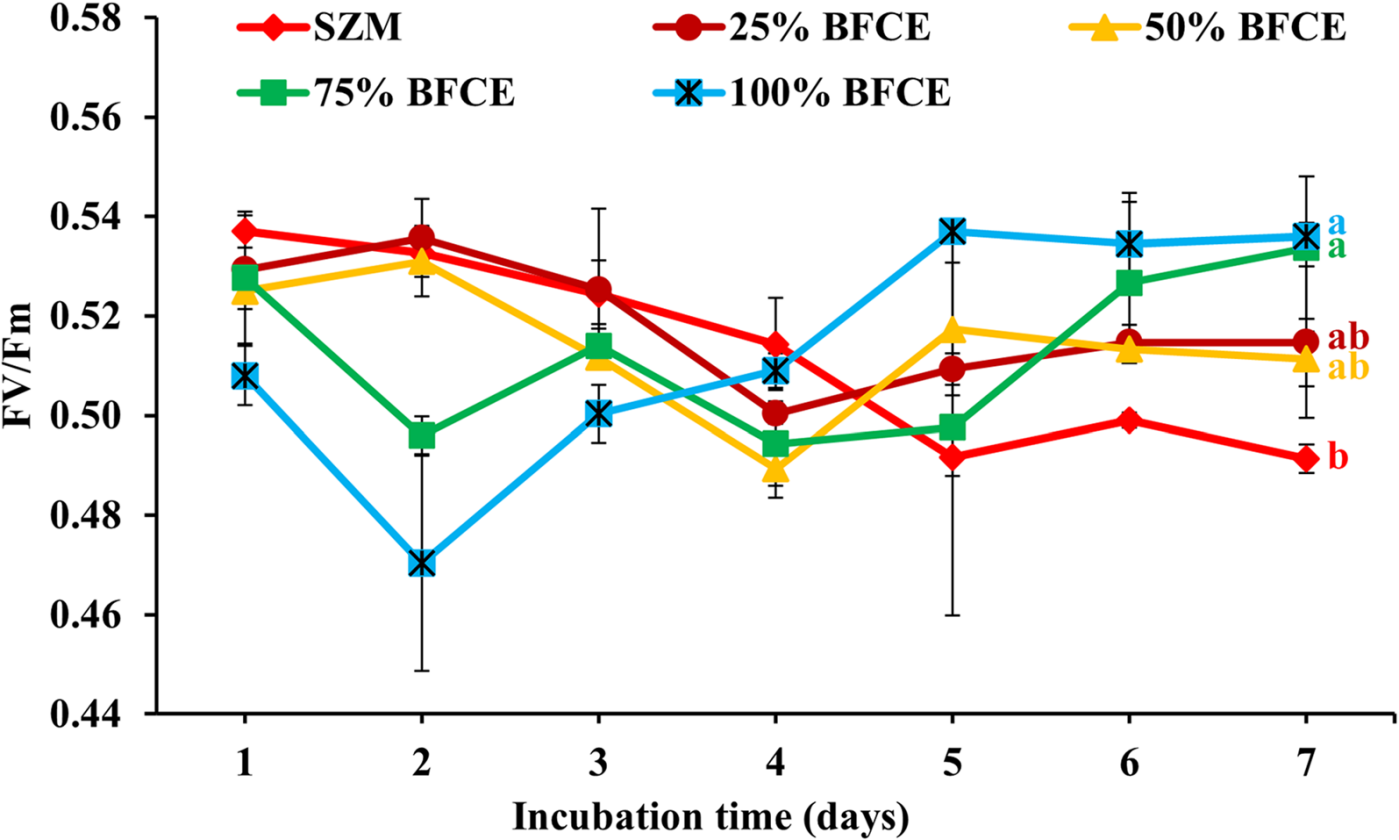
Optimal quantum yield (Fv/Fm) in *S. platensis* ± SE cultivated in the SZM and BFCE treatments

In this experiment, the photosynthetic activity of *S. platensis* grown in both standard zarrouk’s medium (SZM) and BFCE was measured every day to assess the impact of BFCE on photosynthesis performance. Richmond et al. [15] reported that the Fv/Fm values of non-stressed green microalgae range between 0.7 and 0.8, depending on environmental and culturing conditions. However, the values of the cyanobacterial cell range between 0.4 and 0.6 as phycobiliproteins interfere with the fluorescence signal of the chlorophylls inside the cell [16, 17]. In this experiment, Fv/Fm values of all treatments were within the acceptable ranges compared to the SZM, but they showed a decrease to a range below 0.50 in both treatments 75% and 100% on the 2^nd^ day of the experiment due to the stress induced by high concentrations of BFCE at the beginning of the experiment. Later, the alga adapted to this change in the medium nutrient content for 75% and 100% treatments. At the end of the experiment on the 7th day, the lowest activity was in the SZM, and this may be due to low nutrient availability caused by *S. platensis* utilization or due to a self-shading effect caused at the end of log growth phase, which effect on photosynthetic potential [18, 19], while 75% and 100% were the highest values, as shown in Fig. 1 and Additional file 1: Fig. S1.

Numerous studies have shown that Fv/Fm values obviously change by stress conditions during growth, such as high light intensity, nutrient deficiency, oxygen, radiation, and excess heavy or toxic elements [20–23].

#### Evaluation of growth by optical density

**Fig. 2:**
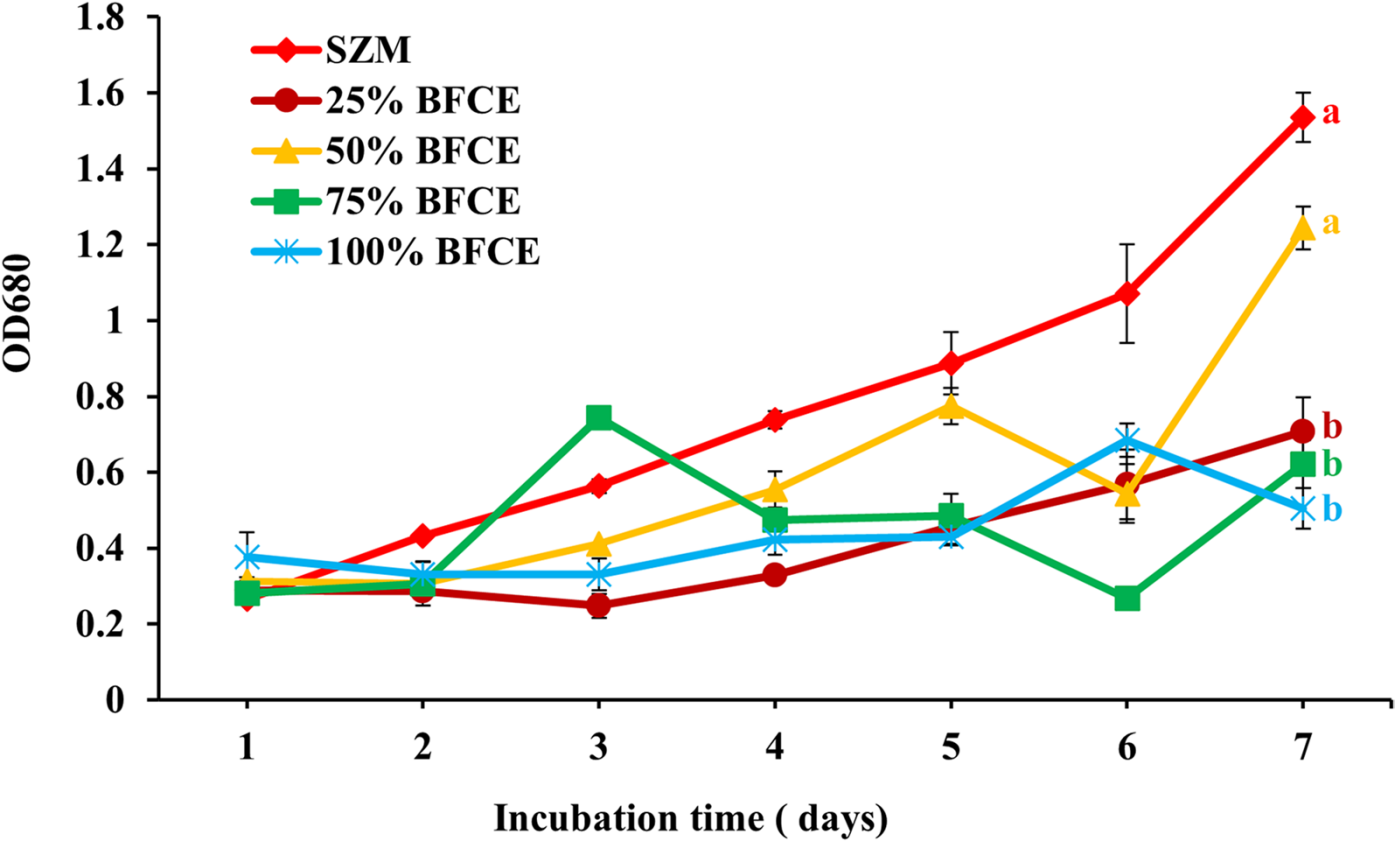
Optical density of *S. platensis* ± SE cultivated in the SZM and BFCE treatments

*S. platensis* growth rate increases in all concentrations until the fifth day, but the growth decreases in the 75% BFCE on the sixth day and then increases again. Similarly, the growth declined in the 100% BFCE (Fig. 2). The high BFCE concentration resulted in lower biomass accumulation due to insufficient nutrients. Furthermore, the growth of 50% BFCE treatment (0.3–1.24) was closer to the SZM (0.2–1.53) but less than it, with an unclear sudden decline in the 6th day followed by growth recovery by the 7th day, which may be due to the non-uniform culture density causing *S. platensis* clumps.

#### Estimation of dry-weight biomass

**Fig. 3:**
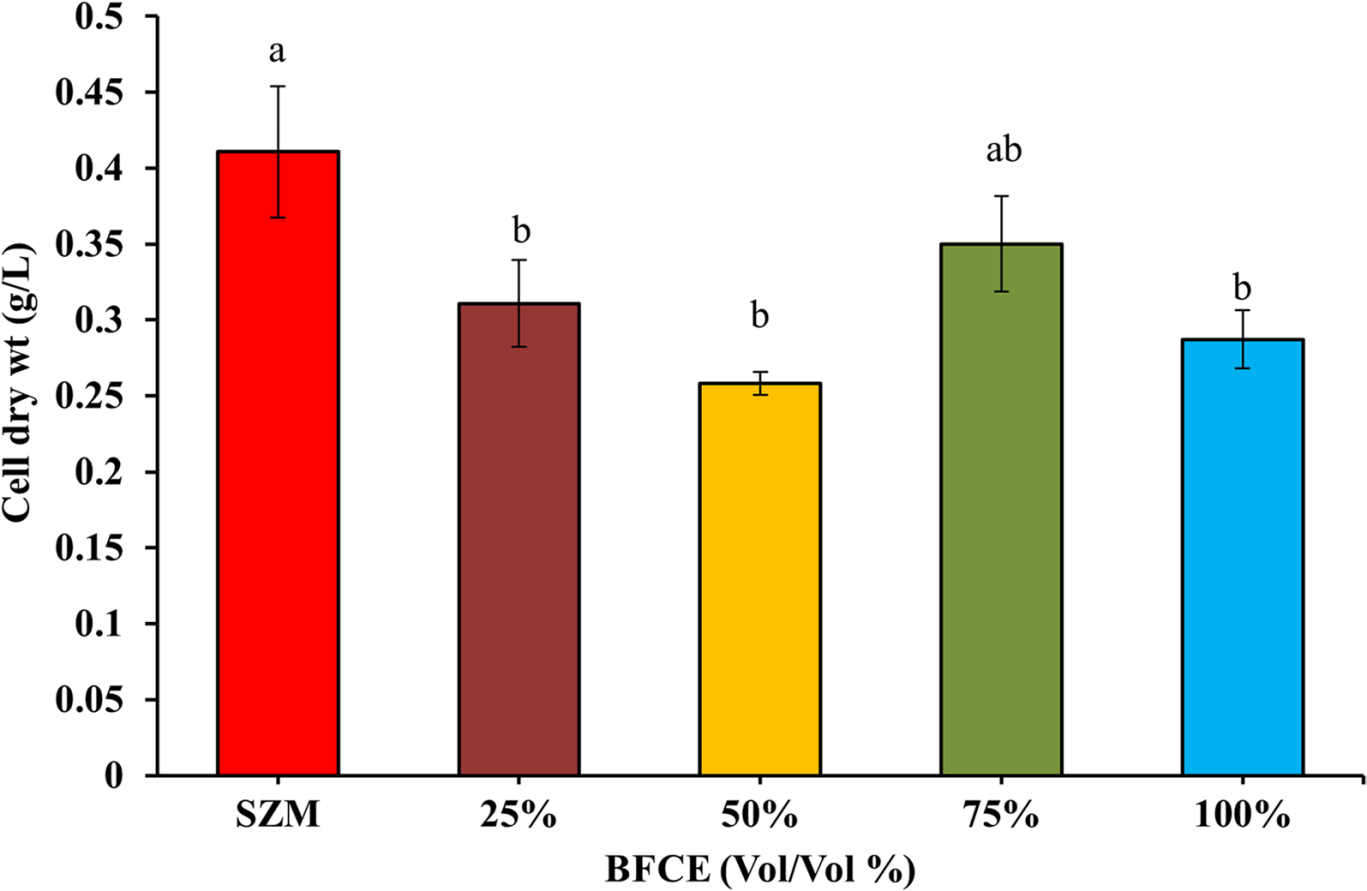
Dry weight of *S. platensis* ± SE cultivated in the SZM and BFCE treatments

As shown in Fig. 3, the highest dry weight determined of *S. platensis* was in SZM (0.41 ± 0.04 g/L), followed by 75% BFCE (0.34 ± 0.03 g/L), while the minimum dry biomass was at 50% BFCE (0.25 ± 0.007 g/L). The variation in dry cell weight was significantly decreased (p ≤ 0.05) in different concentrations of BFCE except for 75%, which gave a non-significant decrease compared to the SZM.

#### Chlorophyll-*a*, *b* and carotenoids determination

**Fig. 4:**
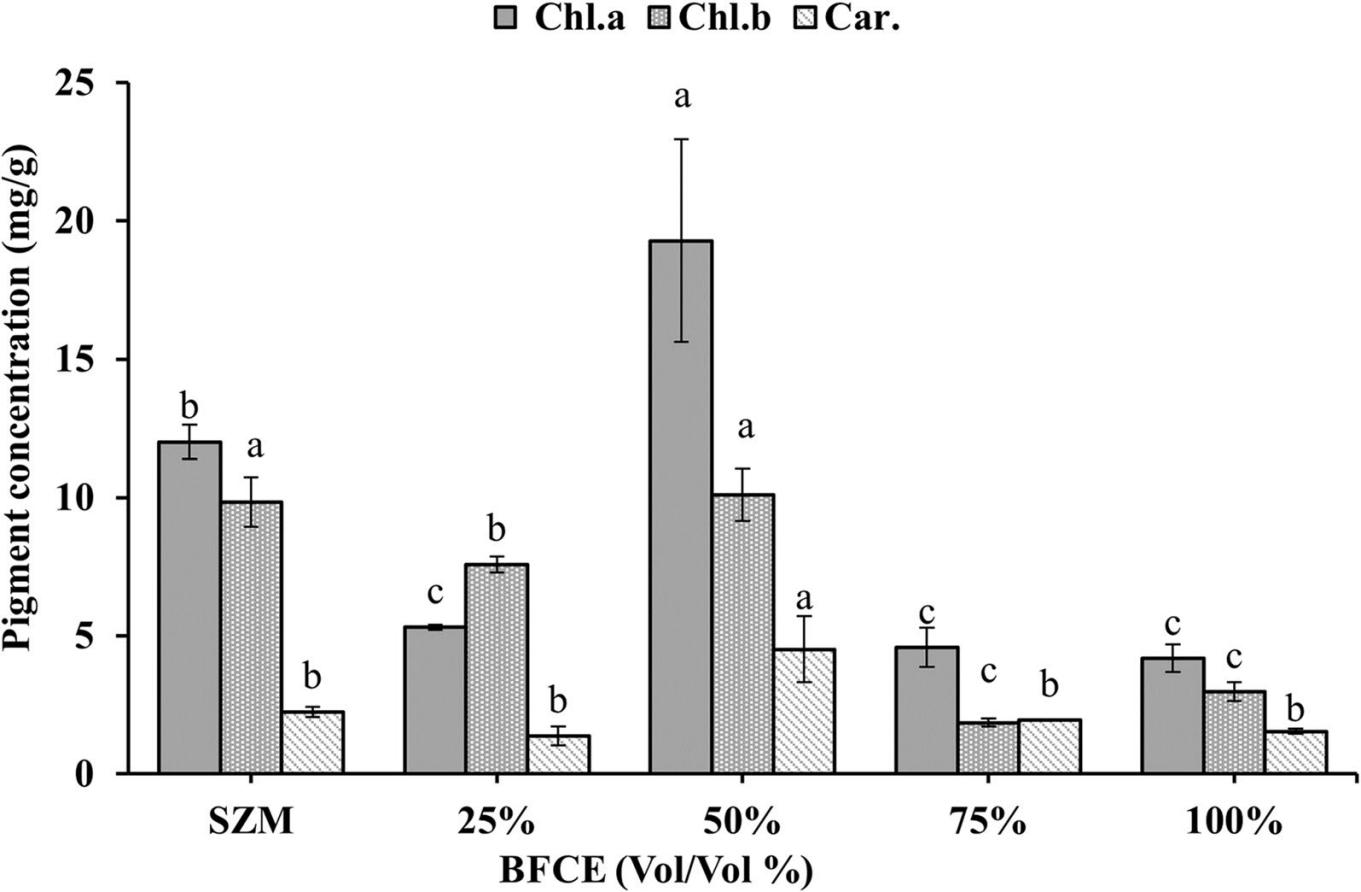
Photosynthetic pigments in *S. platensis* ± SE cultivated in the SZM and BFCE treatments

The chlorophyll-*a* concentration is directly related to biomass concentration and protein content. The maximum chlorophyll-*a* concentration (19.284 ± 3.6 mg/g) was obtained in 50% BFCE, followed by the SZM (12.0 ± 0.6 mg/g), while the concentration of chlorophyll-*b* in SZM and 50% BFCE was almost similar as shown in Fig. 4. All pigment concentrations declined when the BFCE concentration was increased to 100%. Further addition of BFCE up to 50% resulted in a significant increase in carotenoids content (4.5 ± 1.19 mg/g) compared to SZM (2.24 ± 0.18 mg/g), but other concentrations showed non-significant differences with SZM.

#### Estimation of protein

**Fig. 5:**
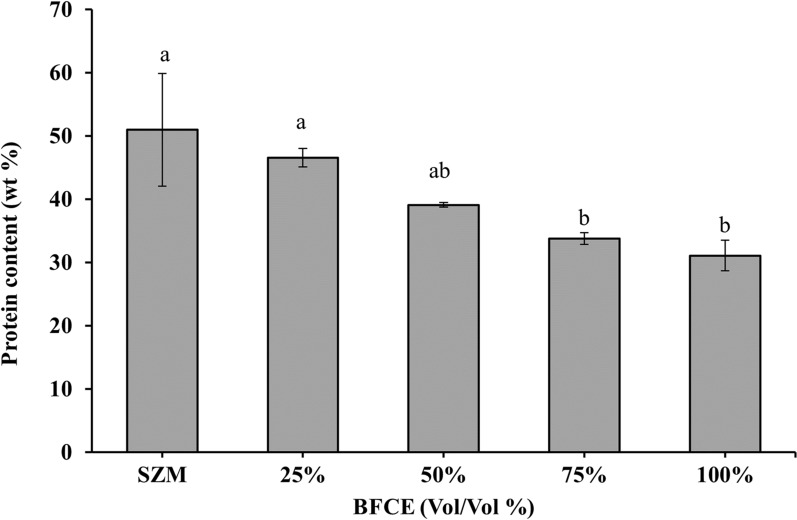
Protein content in *S. platensis* ± SE cultivated in the SZM and BFCE treatments

The standard nutritional profile of *S. platensis* is proteins (50–70%), carbohydrates (15–25%), lipids (6–8%) and minerals (7–13%). These significant components' percentages alter with the cultivation conditions of the alga [12]. In agreeing with these ranges, the highest protein concentration in this study was 50.98 ± 8.9% in SZM, followed by 25% (46.56 ± 1.4%), then 50% (39.11 ± 0.36%). In contrast, at 100% of BFCE, the biomass contained in lower protein content equals 31.1 ± 2.4% (Fig. 5).

In a relevant study [24], the protein content in *S. platensis* biomass cultivated in raw piggery effluents medium was 45.31%, while supplementation with ferrous sulfate caused an increase in the protein content up to 55.15%. Similarly, direct cultivation of *S. platensis* at different concentrations of BFCE had a significant negative impact on the protein content at p ≤ 0.05 by increasing the BFCE concentrations. Because the BFCE is limited in nutrient content compared to the standard growth medium, and as a result, these conditions cause a nutrient deficiency, especially nitrogen concentration, which affects the protein content. In agreement, Uslu et al. [25] concluded that the lack of NaNO_3_ limited protein synthesis, which resulted in a protein productivity decline.

#### Total carbohydrate estimation

**Fig. 6:**
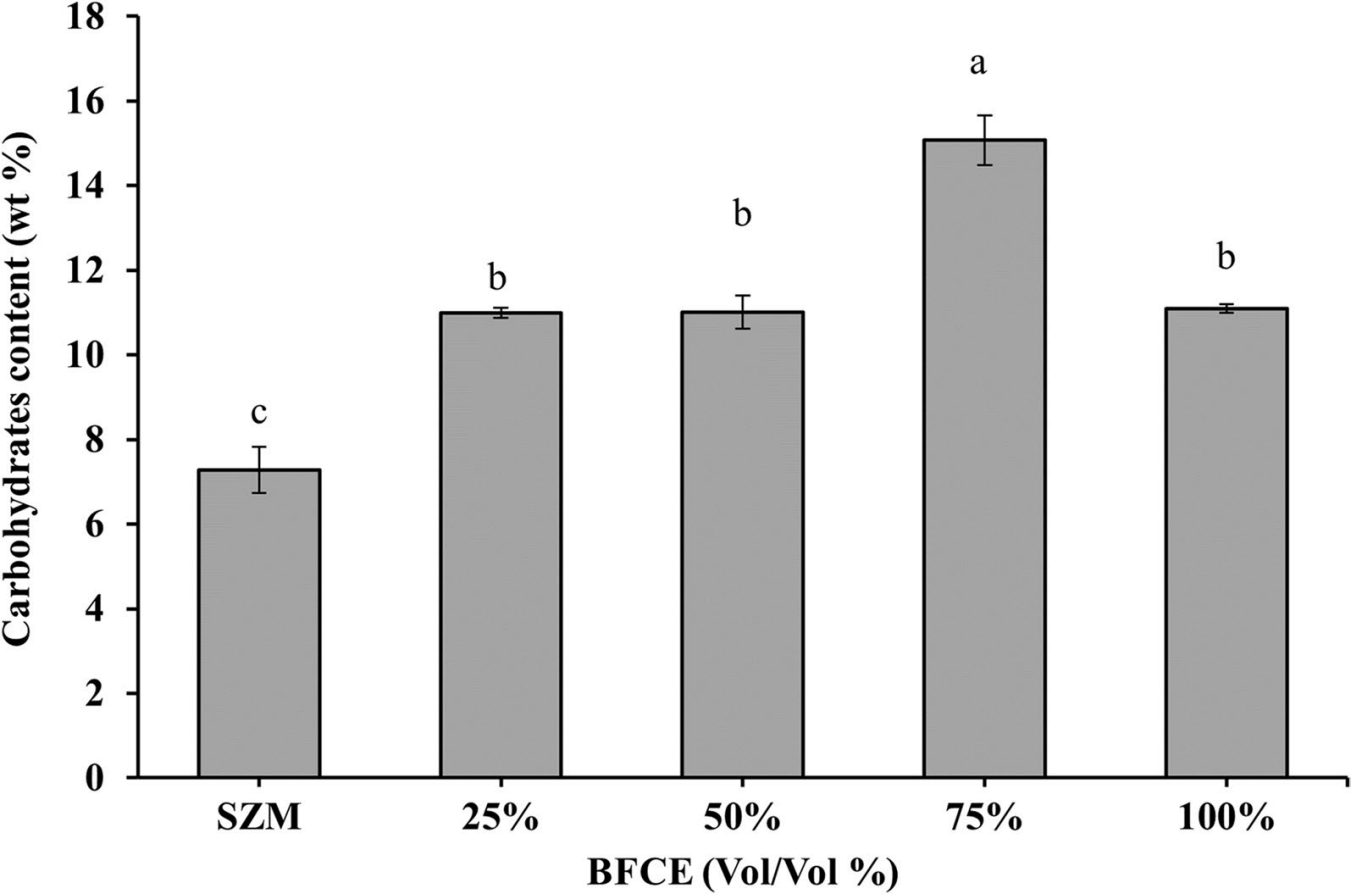
Carbohydrates content in *S. platensis* ± SE cultivated in the SZM and BFCE treatments

The carbohydrate content in *S. platensis* culture, which was cultivated in this experiment, showed a generally increasing trend, as shown in Fig. 6; all treatments of 75%, 50%, 25%, and 100% BFCE produced higher carbohydrate contents (15.07 ± 0.5%, 11 ± 0.5%, 10.9 ± 0.12% and 11.0 ± 0.1%, respectively) compared to SZM which had only 7.28 ± 0.54% of carbohydrates. This trend in the induction of carbohydrates content agreed with the results of growing different microalgae such as *Chlorella vulgaris*, *Phaeodactylum tricornutum*, and *Micractinium pusillum* in other mixotrophic growth conditions [26–29].

The carbohydrate content was enhanced by increasing the BFCE concentrations to 75% (15.07 ± 0.58%), which significantly differs from SZM (7.28 ± 0.5%). In comparison, the pure BFCE 100% led to decreased carbohydrate content. In SZM, *S. platensis* grows autotrophically using only carbon dioxide and sodium bicarbonates as the sole carbon source for carbohydrate accumulation. However, in SZM supplemented with BFCE with different ratios, *S. platensis* grew mixotrophically_,_ simultaneously accumulating more carbohydrates from other carbon sources in BFCE.

#### Total lipid estimation

**Fig. 7:**
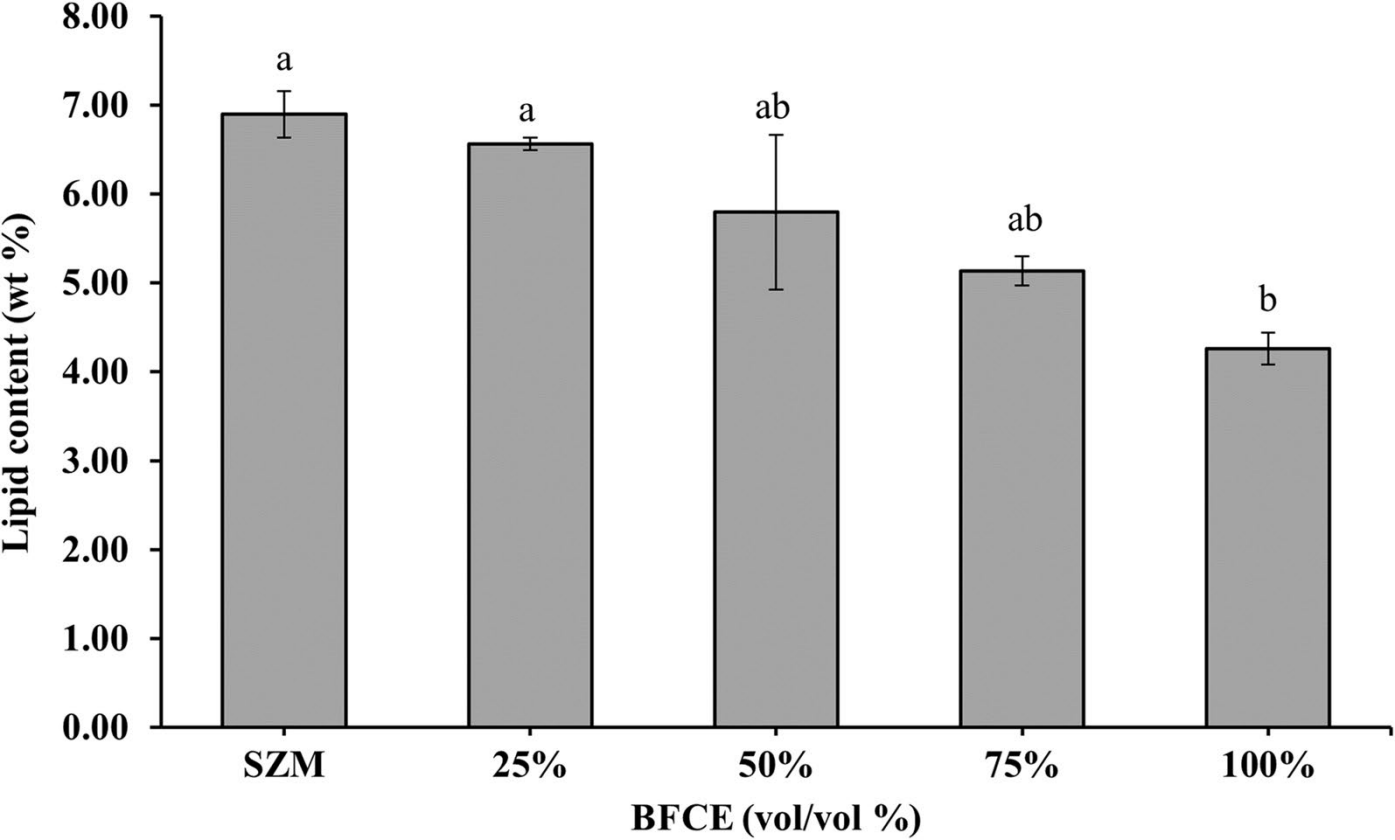
Lipid content in *S. platensis* ± SE cultivated in the SZM and BFCE treatments

The lipid content represented in Fig. 7 showed that the SZM had the highest value up to 8.14 ± 0.26%, while 25% of BFCE resulted in only 6.56 ± 0.07% lipids. In the same reversal relationship between BFCE and lipids content, the 50%, 75% and 100% BFCE concentrations produced 7.62 ± 0.8%, 5.13 ± 0.1%, and 4.26 ± 0.18%, respectively.

In general, *S. platensis* lipid contents showed relatively low proportions; therefore, higher BFCE concentrations showed no remarkably significant impact on lipid accumulation in the lyophilized biomass. These results were lower than that obtained by Chang Y, et al. [30] (17.2–19.8%), they cultivated *S. platensis* on synthetic human urine for biomass production and nutrient removal. Also, Li et al. [31] obtained the same results when studying nitrogen limitation's effect on *S. platensis* performance cultivated mixotrophically.

### Incremental waste acclimatization experiment

#### Photosynthetic activity

**Fig. 8:**
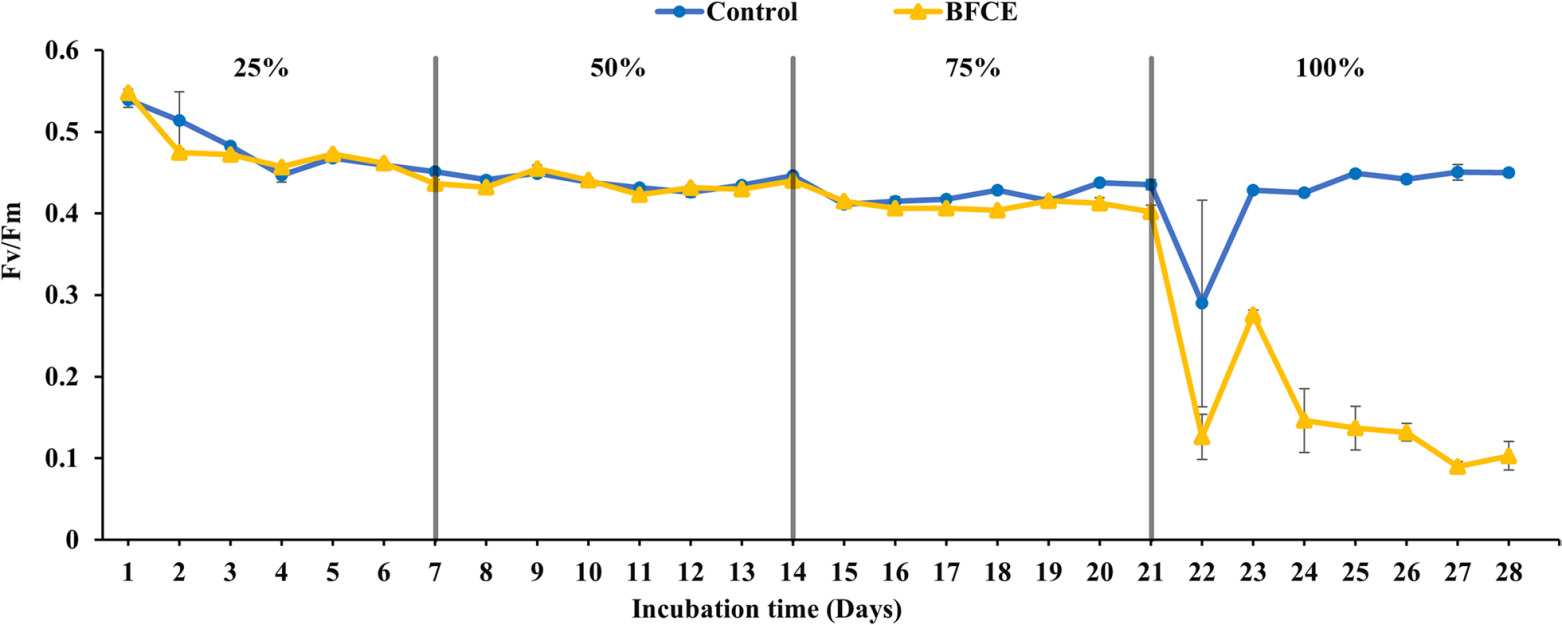
Optimal quantum yield (Fv/Fm) in *S. platensis* ± SE cultivated in the SZM and BFCE treatments

The functional consequences of changes in the maximum photochemical efficiency of photosystem II inferred by the Fv/Fm values of dark-adapted cultures and a decline in the value of this parameter are thought to be a sign of environmental stress or nutritional depletions [32]. For instance, in *S. platensis*, the electron transport chain in PS II was reported to be damaged by the effect of increasing salinity [33]. At the start of the experiment, in both treatments, the Fv/Fm was 0.5, then decreased to 0.4 during all cultivation periods. In addition, the Fv/Fm values of BFCE from 25 to 75% were consistent with the values of SZM, confirming that *S. platensis* can adapt to grow up to 75% BFCE. On the other hand, it decreased dramatically from 0.5 to approximately 0.1 at 100% BFCE. Increasing the BFCE to 100% revealed stress in these culturing conditions causing a decline in the photosynthetic efficiency and adaption potential, as illustrated in Fig. 8. Moreover, this is due to the BFCE containing restricted amounts of nutrients, especially nitrogen and phosphate, compared to the standard medium for *S. platensis* growth. Consistent with Liu et al. [34], they observed a reduction in Fv/Fm values when microalga *Phaeodactylum tricornutum* was grown in a mixotrophic mode of nutrition. Also, Tang et al. [35] reported that the Fv/Fm of *Scytonema javanicum* during their experiments was significantly decreased by salinity stress during growth.

The high concentrations of BFCE decreased the *S. platensis* growth rate, photosynthetic pigment, protein, carbohydrates, and lipid contents. Based on the naked eye optical observation, the culture colour turned from blue-green to yellow-green by the last run when BFCE totally replaced SZM. This could be due to the insufficient nitrogen content in the cultivation medium, which makes *S. platensis* utilize the pigments as a nitrogen storage material for alternative protein synthesis. These results are harmonious with many studies [36, 37].

#### Evaluation of growth optical density

**Fig. 9:**
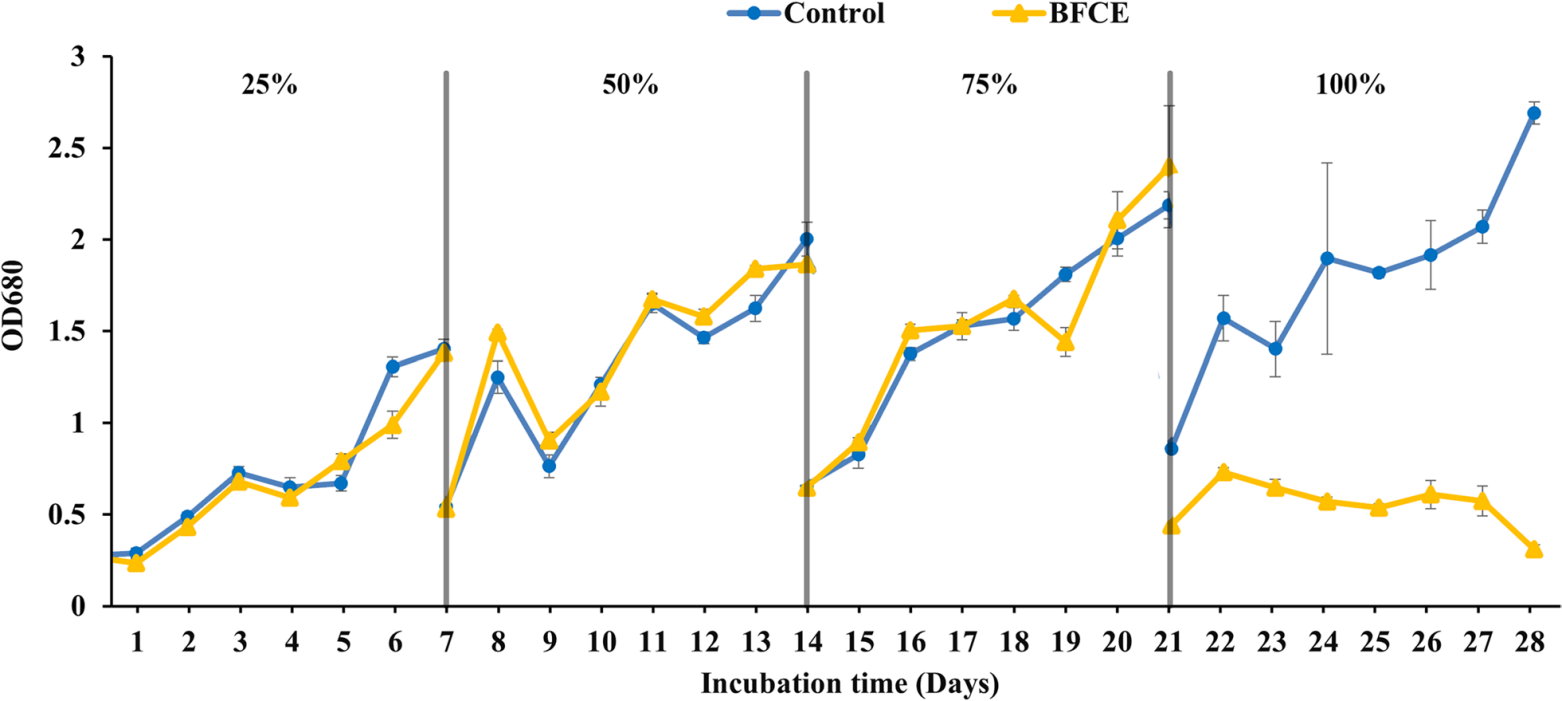
Optical density of *S. platensis* ± SE cultivated in the SZM and BFCE treatments

The behaviour of *S. platensis* in SZM and BFCE-supplemented media was an almost similar trend (Fig. 9), indicating that *S. platensis* had adapted to grow on specific concentrations of BFCE up to 75%. On the other hand, there was a significant decrease at 100% BFCE (0.31 ± 0.02) as shown in Additional file 1: Fig. S2, which could be attributed to the limitation effect on the photosynthetic rate of *S. platensis* showing that combining nutrients from SZM and BFCE is better for *S. platensis* biomass production on a large scale than BFCE alone, which is limited in its nutritional components.

#### Estimation of dry-weight biomass

**Fig. 10:**
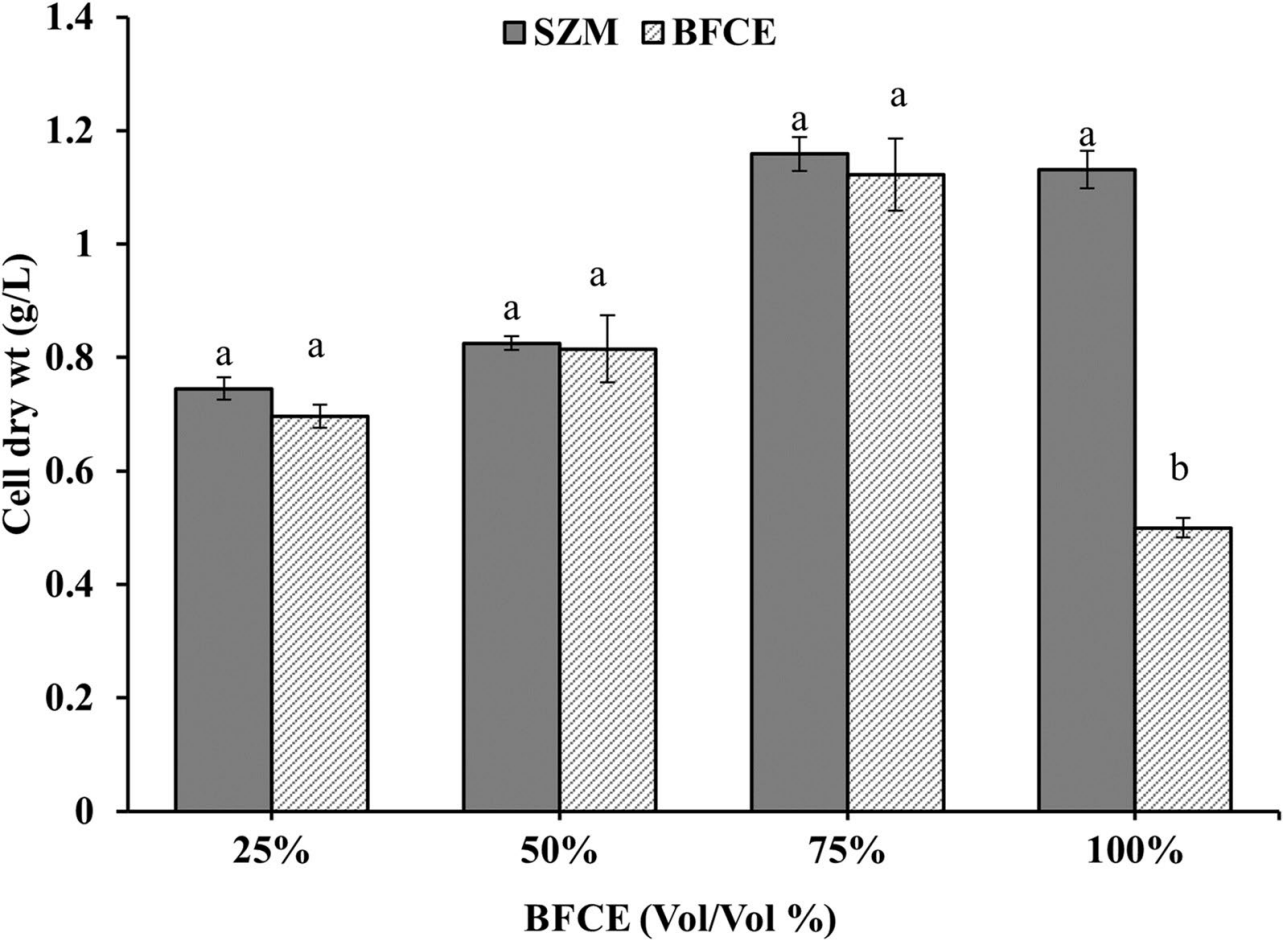
Dry weight of *S. platensis* ± SE cultivated in the SZM and BFCE treatments

As shown in Fig. 10, The cell dry weight of *S. platensis* increased with the gradual rising of BFCE up to 75% (1.122 ± 0.063 g/L) with a non-significant difference. Excessive BFCE concentrations, up to 100%, had a remarkable negative impact on the cell dry weight of *S. platensis* (0.49 ± 0.034 g/L), revealing that *S. platensis* dry weight increasing trend is similar to the optical density, up to 75% BFCE.

#### Chlorophyll-*a*, *b* determination

**Fig. 11:**
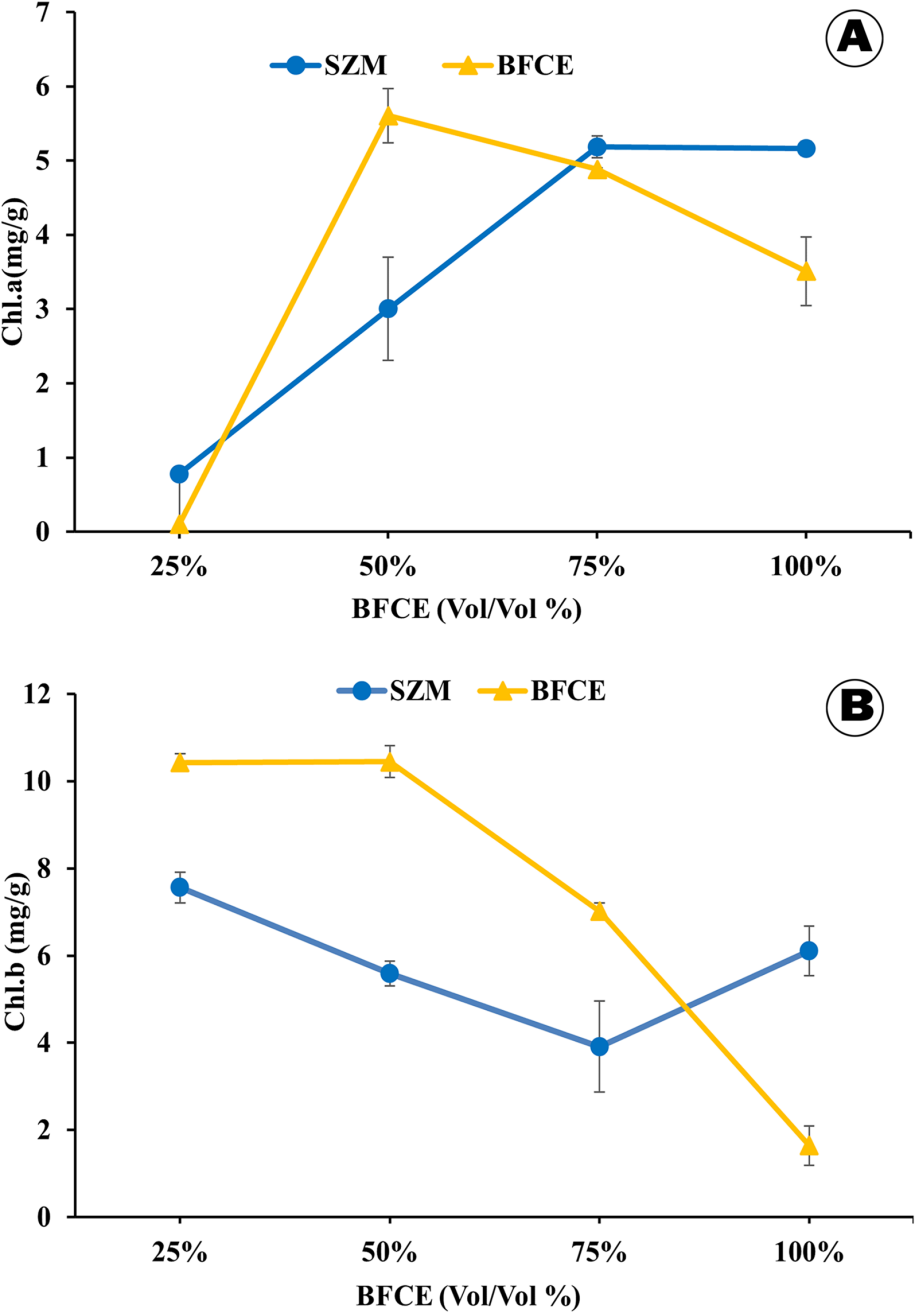
Photosynthetic pigments in *S. platensis* ± SE cultivated in the SZM and BFCE treatments. **A**: Chlorophyll-a in mg/g dry weight and **B** Chlorophyll-b in mg/g dry weight

The chlorophyll content of *S. platensis* biomass depends on medium composition, particularly the kind and amount of nitrogen [38, 39]. The chlorophyll-*a* increased in *S. platensis* cultivated in SZM during the entire experiment period. However, it raised to (5.6 ± 0.3 mg/g) when the BFCE concentration was increased to 50%, but it dropped again when the concentration was raised to 100% (3.5 ± 0.4 mg/g). Moreover, the chlorophyll-b increased when using 25% and 50% BFCE over the SZM, then decreased again in 100% BFCE concentration (Fig. 11). In this design the carotenoids not detected in BFCE expect at 100% BFCE concentration (0.57 mg/g), while in SZM the concentration increased from 0.37 to 1.70 mg/g during cultivation period. The feasibility of using low BFCE concentrations along with SZM in *S. platensis* cultivation was significant because the growth, photosynthetic activity, dry weight, and photosynthetic pigment were not affected. While at higher concentrations of BFCE, the cell growth and photosynthetic activity were significantly decreased, consequently causing a decrease in chlorophyll contents. Furthermore, similar results were recorded and concluded that pigment content in *S. platensis* was lower in mixotrophic than autotrophic conditions [31].

#### Estimation of protein

**Fig. 12:**
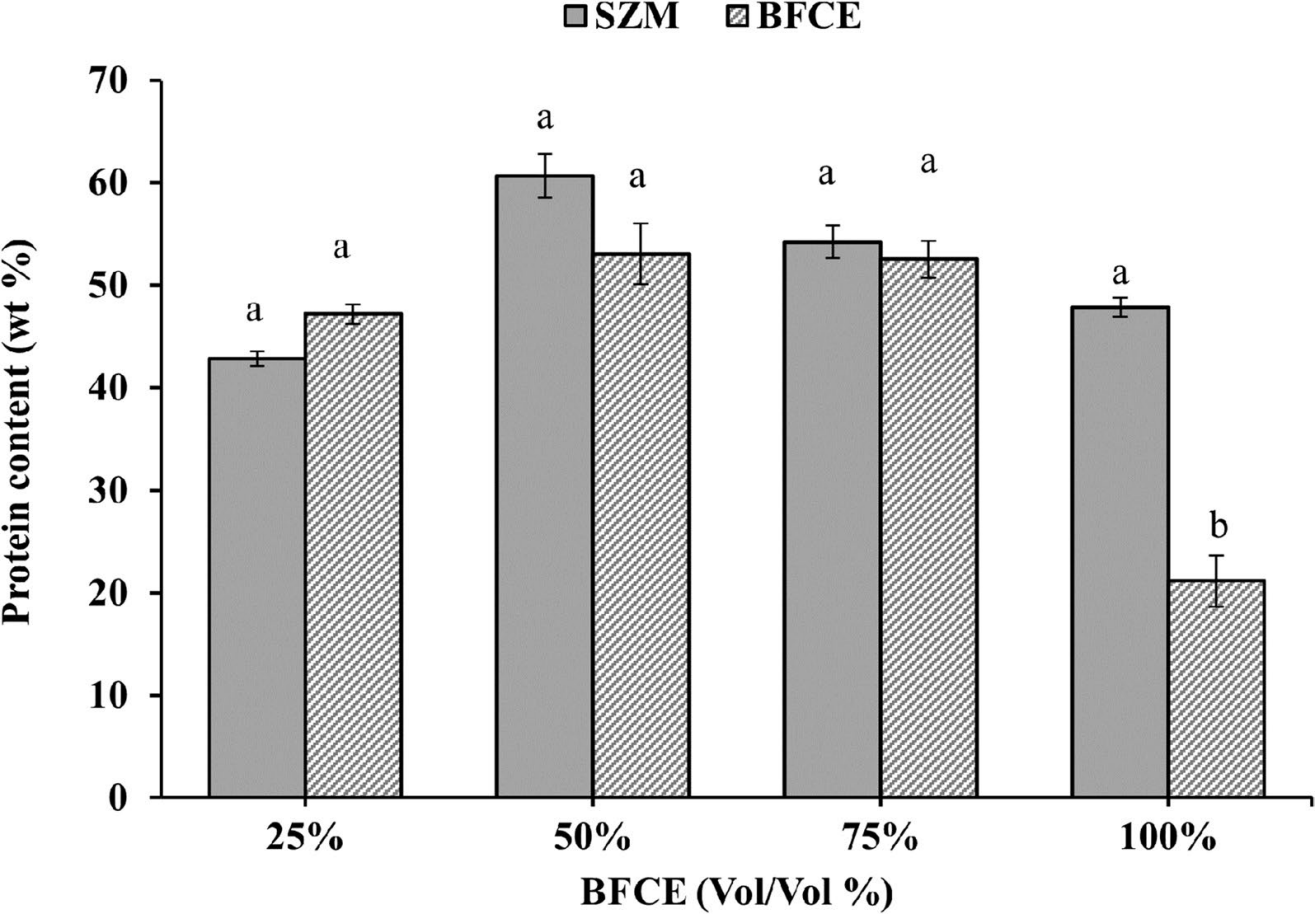
Protein content in *S. platensis* ± SE cultivated in the SZM and BFCE treatments

According to the data presented in Fig. 12, the protein content increased from 47.18 ± 0.9% to 52.54 ± 1.7% in treatments from 25 to 75% BFCE with minor variation compared to the SZM (p ≤ 0.05). However, a considerable reduction in protein content was observed at 100% BFCE concentration. At the same time, the SZM varied from 42.84% protein in the 1^st^ week of the experiment to 60.66%, which was the maximum peak value of protein production, in the 2nd week.

It is known that nitrogen is one of the limiting factors that affect the growth and chemical composition of cyanobacteria [40] and the accumulation of nitrogen-rich biomolecules such as protein in microalgal biomass is directly related to nitrogen availability in the nutrient medium [41].

As mentioned, BFCE contains less nitrogen than the SZM used for *S. platensis*. Gradually supplying the SZM with BFCE led to an increase in protein content. However, at 100% BFCE, a significant protein content reduction was noticed at only 21.16% compared to SZM, which produced 47.80% protein. These results were consistent with Piorreck et al. [42], they observed that the increase in the nitrogen level supports an increase in biomass, protein content, and chlorophyll. In agreement with these results, the *Spirulina* maxima was grown on fermented cattle, and poultry manure with high nitrogen showed a 60.1% and 71.8% protein content, respectively [43]. Considering the criteria of economic feasibility studies, it is clear that the optimum use of BFCE as additives to the growth medium is 75% because the decrease in protein production is not significantly different from the SZM.

#### Total carbohydrates determination

**Fig. 13:**
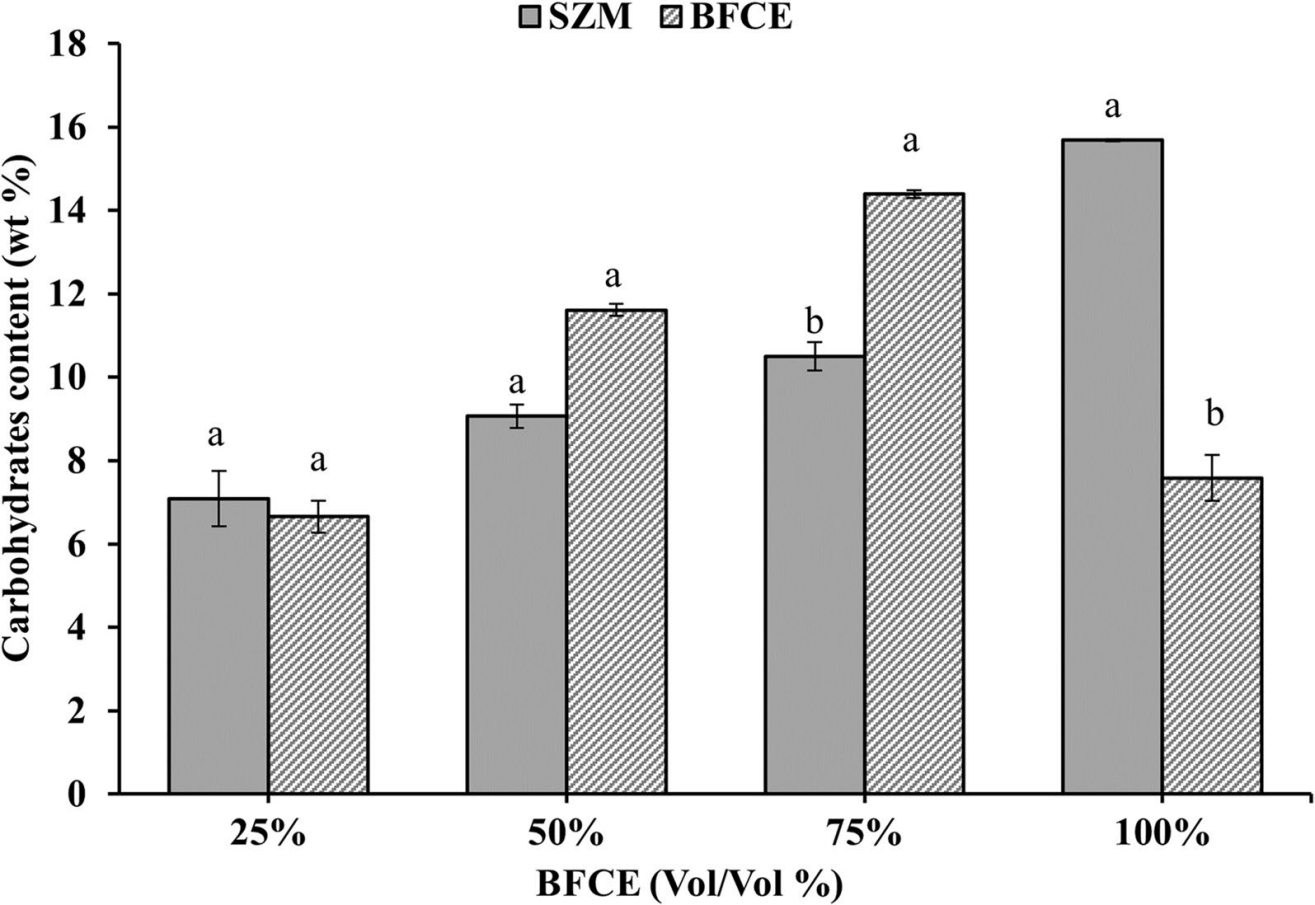
Carbohydrates content in *S. platensis* ± SE cultivated in the SZM and BFCE treatments

Carbohydrates content increased from 6.65 ± 0.3% to 14.39 ± 0.09% in treatments 25% to 75% BFCE with an insignificant difference (p ≤ 0.05) compared to SZM (Fig. 13). Moreover, it revealed a considerable reduction in the carbohydrates content at 100% BFCE concentration equal to 7.58 ± 0.5%, which may be attributed to the reduction in growth rate due to the low availability of nutrients with the increase in the BFCE concentration in the culture medium.

The enhanced carbohydrates content in *S. platensis* may be attributed to the utilization of organic carbon in the BFCE as a carbon source in addition to photosynthesis. Likewise, a similar study reported an improving in carbohydrate content in *S. platensis* growing on tofu wastewater enriched with various organic carbon [44].

#### Total lipid determination

**Fig. 14:**
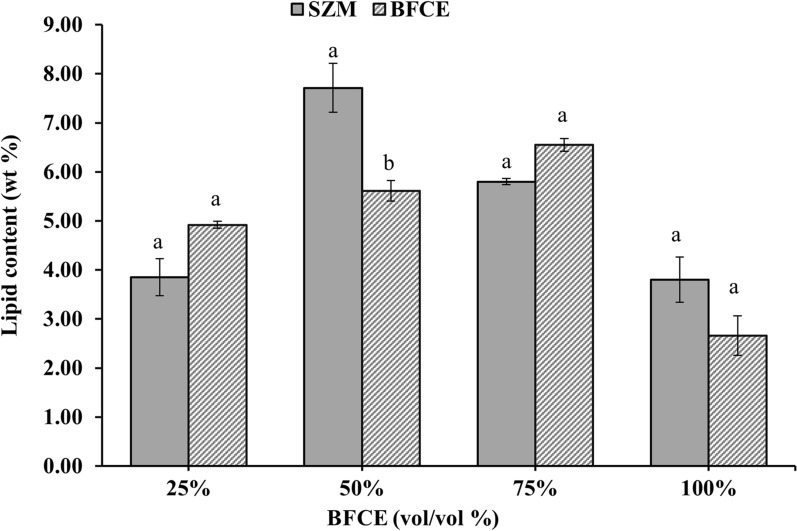
Lipid content in *S. platensis* ± SE cultivated in the SZM and BFCE treatments

The lipid content increased from 4.92 ± 0.07% to 6.55 ± 0.13% in treatments 25% and 75% of BFCE, which was almost similar to that of SZM (3.85 ± 0.38% to 7.71 ± 0.5%). In contrast, 100% BFCE resulted in only 2.66 ± 0.4%. The lipid content of the biomass was not significantly affected by the different concentrations of BFCE (Fig. 14). The difference compared to the SZM was insignificant at p-value ≤ 0.05, in general.

In another study investigating the effect of nitrogen concentration as NaNO_3_ on the lipid accumulation in *S. platensis*, the maximum lipid production was 4.7% at 15 °C in a nitrogen-free medium. In contrast, the best growth and lipid production was at 35 °C and 1.25 g/L of sodium nitrate [45].

In general, a slight difference between all SZM during the four weeks of the experiments is owing to the variation in the inoculum dry weight (g/L) which coincide with Pelizer et al. [46], they stated that the best inoculum age was six days with 50 mg/L of concentration and changing this ratio will impact on the final dry weight and biochemical composition of the biomass.

### Optimization of growth parameters using central composite design (CCD)

Factors such as NaNO_3_, NaHCO_3_, BFCE and PH were predicted to affect the *S. platensis* growth and protein yield. Moreover, NaNO_3_ and NaHCO_3_ are the main costly elements in the SZM and highly considered in the economic feasibility of large-scale production of *S. platensis* while BFCE is a massive sugar industry waste with non-significant application and cheap prices. On the other hand, it is a source of carbonates and still contains impurities from the sugar industry [47] which considered as a valuable nutrient for *S. platensis*. A CCD matrix was performed to detect the appropriate NaNO_3_, NaHCO_3_, BFCE and PH controlling the maximum biomass and protein yield. Biomass growth is expressed as dry weight (g/L) and protein yield (%). The optical density and photosynthetic activity (Fv/Fm) of *S. platensis* cultivated using CCD matrix are shown in Additional file 1: Table S1. The matrix and responses of the design are shown in Table 2 and Additional file 1: Fig. S3. The maximum protein was 54.40%, and biomass dry weight was 0.74 g/L in runs number 2 and 15, respectively. In run number 2 when the NaHCO_3_ was reduced to 25% of the initial content (4.2 g/L), the protein content and dry biomass were nearly the same as SZM. This contradicts the conclusion that appeared with Raoof et al. [48] reported that the reduction in the bicarbonate concentration to 4 g/L in the formulated medium led to a significant decrease in biomass, chlorophyll and protein content. In run number 15, when the NaNO_3_ reduced to 1.075 g/L led to a decrease in protein content compared to the SZM and the response surface quadratic model for biomass yield and protein concentration are tabulated in Table 3.

**Table 2:** Matrix and its corresponding responses for the CCD experiment

Run	Variables				Responses	
	NaNO_3_ (g/L)	NaHCO_3_ (g/L)	BFCE (%)	pH	Biomass dry weight (g/L)	Protein (%)
1	1.075	4.2	0.375	8.75	0.37	37.28
**2**	2.025	4.2	0.375	8.75	0.55	54.40
3	1.075	12.6	0.375	8.75	0.52	46.27
4	2.025	12.6	0.375	8.75	0.65	35.18
5	1.075	4.2	0.625	8.75	0.60	34.18
6	2.025	4.2	0.625	8.75	0.59	42.73
7	1.075	12.6	0.625	8.75	0.61	38.33
8	2.025	12.6	0.625	8.75	0.40	54.20
9	1.075	4.2	0.375	10.25	0.48	39.33
10	2.025	4.2	0.375	10.25	0.52	38.31
11	1.075	12.6	0.375	10.25	0.47	50.89
12	2.025	12.6	0.375	10.25	0.58	42.81
13	1.075	4.2	0.625	10.25	0.74	30.72
14	2.025	4.2	0.625	10.25	0.64	42.95
**15**	1.075	12.6	0.625	10.25	0.74	41.79
16	2.025	12.6	0.625	10.25	0.68	41.77
17	0.6	8.4	0.5	9.5	0.61	46.82
18	2.5	8.4	0.5	9.5	0.62	46.16
19	1.55	0.0	0.5	9.5	0.56	31.14
20	1.55	16.8	0.5	9.5	0.74	43.58
21	1.55	8.4	0.25	9.5	0.59	50.42
22	1.55	8.4	0.75	9.5	0.69	44.94
23	1.55	8.4	0.5	8	0.63	44.94
24	1.55	8.4	0.5	11	0.74	35.74
25	1.55	8.4	0.5	9.5	0.58	45.04
26	1.55	8.4	0.5	9.5	0.57	42.34
27	1.55	8.4	0.5	9.5	0.51	44.07
28	1.55	8.4	0.5	9.5	0.55	41.89
29	1.55	8.4	0.5	9.5	0.52	45.32
30	1.55	8.4	0.5	9.5	0.54	47.80
31	1.55	8.4	0.5	9.5	0.54	43.28
Control	2.5	16.8	0	9	0.60	55.11

**Table 3:** ANOVA table for response surface quadratic model for biomass yield and protein concentration

Variable	Response											
	Protein (%)						Biomass dry weight (g/L)					
Source	Sum of squares	df	Mean square	F value	*p*-value		Sum of squares	df	Mean square	F value	*p*-value	
Model	765.07	14	54.65	2.50	0.0406^a^	Significant	0.1675	10	0.1675	3.57	0.0074^a^	Significant
A-NaNO_3_	43.31	1	43.31	1.98	0.1780		0.0004	1	0.0004	0.088	0.768	
B-NaHCO_3_	131.70	1	131.70	6.03	0.0258^a^	Significant	0.0113	1	0.0113	2.40	0.136	
C-BFCE	34.46	1	34.46	1.58	0.226		0.04	1	0.04	9.98	0.004^a^	Significant
D-pH	43.74	1	43.74	2.00	0.176		0.0258	1	0.0258	5.40	0.030^a^	Significant
A2	0.0312	1	0.0312	0.0014	0.970		0.000733	1	0.000733	0.16	0.69	
B2	144.70	1	144.70	6.63	0.0203^a^	Significant	0.0053	1	0.0053	1.2	0.289	
C2	3.12	1	3.12	0.143	0.710		0.0036	1	0.0036	0.81	0.382	
D2	64.73	1	64.73	2.97	0.104		0.0149	1	0.0149	3.36	0.085	
A * B	101.00	1	101.00	4.63	0.047^a^	Significant	0.0012	1	0.0012	0.26	0.614	
A * C	98.51	1	98.51	4.51	0.049^a^	Significant	0.0441	1	0.0441	9.40	0.0061	
A * D	46.72	1	46.72	2.14	0.162		0.0006	1	0.0006	0.13	0.718	
B * C	24.21	1	24.21	1.11	0.307		0.0121	1	0.0121	2.58	0.123	
B * D	26.42	1	26.42	1.21	0.287		0.0000	1	0.0000	0.005	0.942	
C * D	6.79	1	6.79	0.311	0.584		0.0256	1	0.0256	5.46	0.030^a^	Significant
Residual	349.15	16	21.82					20				
Lack of fit	277.05	10	27.70	2.31	0.1594	Not significant		14		1.76	0.2500	Not significant
Pure Error	72.11	6	12.02					6				
Core total	1114.22	30						30				
R2 = 70.59%			Adjusted R2 = 44.85%				R2 = 72.58%		Adjusted R2 = 48.58%			

^a^Significant value

Analysis of the main effect, quadratic effect and interaction between factors with *p-*value < 0.05 was considered significant, as shown in Table 3.

Second-order polynomial models that predict the biomass dry weight and protein yield as a function of the experimental variable are shown in Eqs. (1 and 2).

For biomass dry weight:

1 $$\begin{aligned} {\text{Y}}_{{1}} &= 0.{543 } + \, 0.00{53}*{\text{A }} + \, 0.0{217}*{\text{B }} + \, 0.0{445}*{\text{C }} + \, 0.0{328}*{\text{D }} \hfill \\ &\quad + \, 0.00{51}*{\text{A2 }} + \, 0.0{137}*{\text{B2 }} + \, 0.0{112}*{\text{C2 }} + \, 0.0{229}*{\text{D2}} - 0.00{84}*{\text{AB}} \hfill \\ & \quad - 0.0{515}*{\text{AC}} - 0.00{51}*{\text{AD}} - 0.0{285}*{\text{BC }} + \, 0.00{15}*{\text{BD }} + \, 0.0{4}0{1}*{\text{CD}} \hfill \\ \end{aligned}$$

For % protein:

2 $$\begin{aligned} {\text{Y2}} & = {44}.{25 } + { 1}.{344}*{\text{A }} + { 2}.{34}*{\text{B}} - {1}.{198}*{\text{C}} - {1}.{35}*{\text{D }} + \, 0.{389}*{\text{A2}} \hfill \\ & \quad - {1}.{89}*{\text{B2 }} + \, 0.{688}*{\text{C2}} - {1}.{147}*{\text{D2}} - {2}.{51}*{\text{AB }} + { 2}.{48}*{\text{AC}} - {1}.{71}*{\text{AD}} \hfill \\ & \quad + { 1}.{23}*{\text{BC }} + \, 0.00{15}*{\text{BD}} - 0.{65}*{\text{CD}} \hfill \\ \end{aligned}$$

From the ANOVA analysis (Table 3), The model is significant for biomass yield and protein concentration, with *p*-value of 0.0074 and 0.04, respectively. The different independent factors with a *p-value* less than 0.05 indicate that they are significant model terms. Regarding biomass yield, the BFCE (C) and pH (D) significantly affected the biomass yield with *p-*values of 0.004 and 0.030, respectively. The interaction of BFCE with pH (CD) also significantly affected the biomass yield with *p-*values of 0.030.

**Fig. 15:**
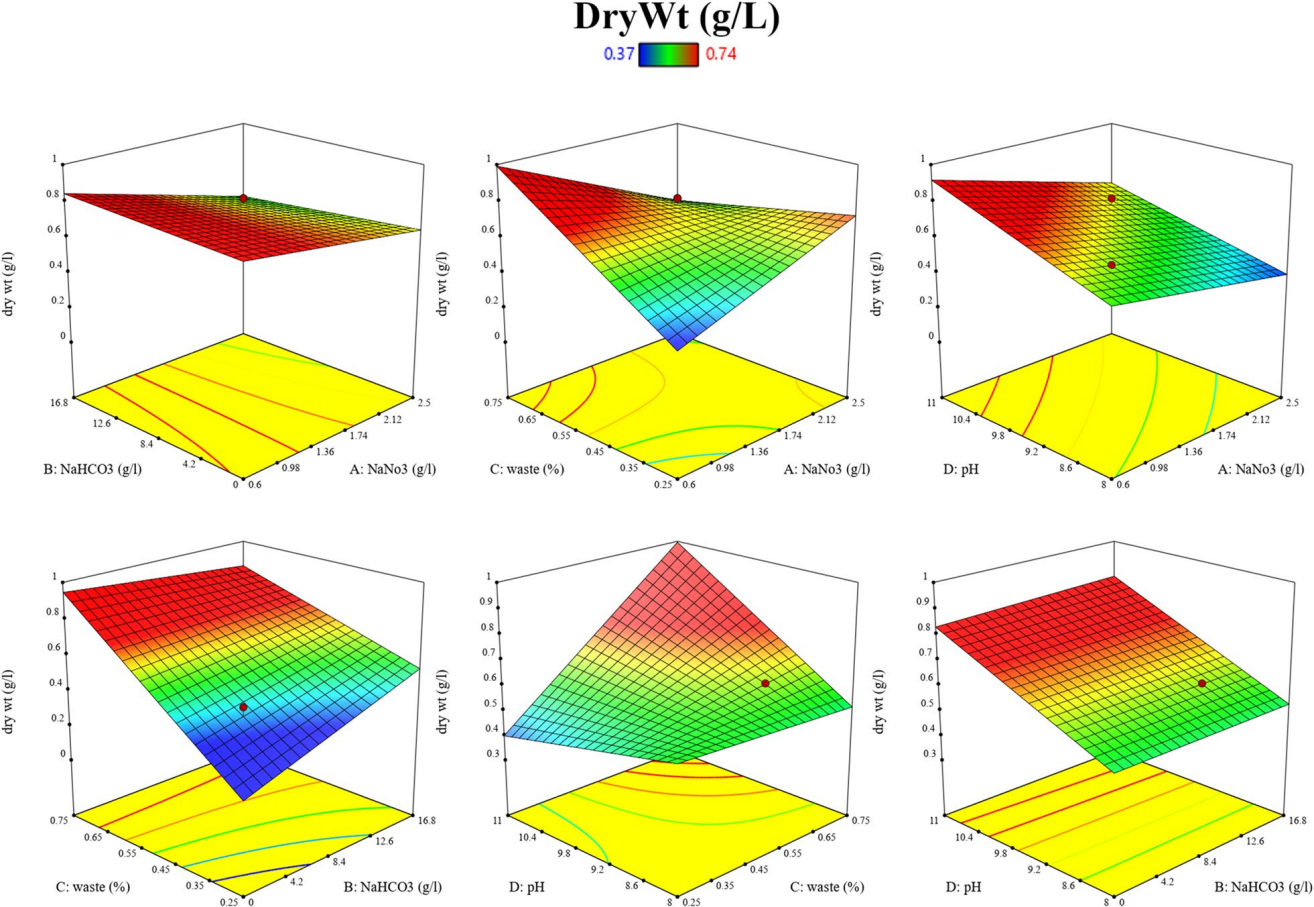
Three-dimensional response surface plot showing the interaction between the tested variables for optimum dry biomass

The interaction between the tested variables on the dry biomass was created by response surface plots (Fig. 15). The deep red colour indicates the direction of the optimum condition for the response. Regarding the interaction between NaNO_3_ (A) and NaHCO_3_ (B), the later optimum concentration was 4.2–16.8 g/L. While in NaNO_3_ (A) and waste (C), the optimum waste concentration was 55–75%. At the same time, NaNO_3_ range was 0.6–1.47 g/L in both interactions. However, in its interaction with waste or pH, its optimum range was 0.6–1.93 g/L, and the optimum ranges for waste and pH were 55–75% and 9.2–11, respectively. Finally, in the interaction between NaHCO_3_ (B) and waste, the optimum range for waste was 65–75%. While in NaHCO_3_ (B) and pH (D), the optimum pH was 9.8–11. Moreover, a NaHCO_3_ concentration of 0–16.8 g/L was the optimum concentration in the interaction with waste and pH.

Regarding the protein, as shown in Table 3, the NaHCO_3_ (B) showed a significant effect with *p*-value of 0.025. The interaction of NaNO_3_ and NaHCO_3_ (AB) and NaNO_3_ and BFCE (AC) and the quadratic terms of NaHCO_3_ (B^2^), also significantly affected the protein yield with *p*-value of 0.047, 0.04 and 0.0203, respectively.

**Fig. 16:**
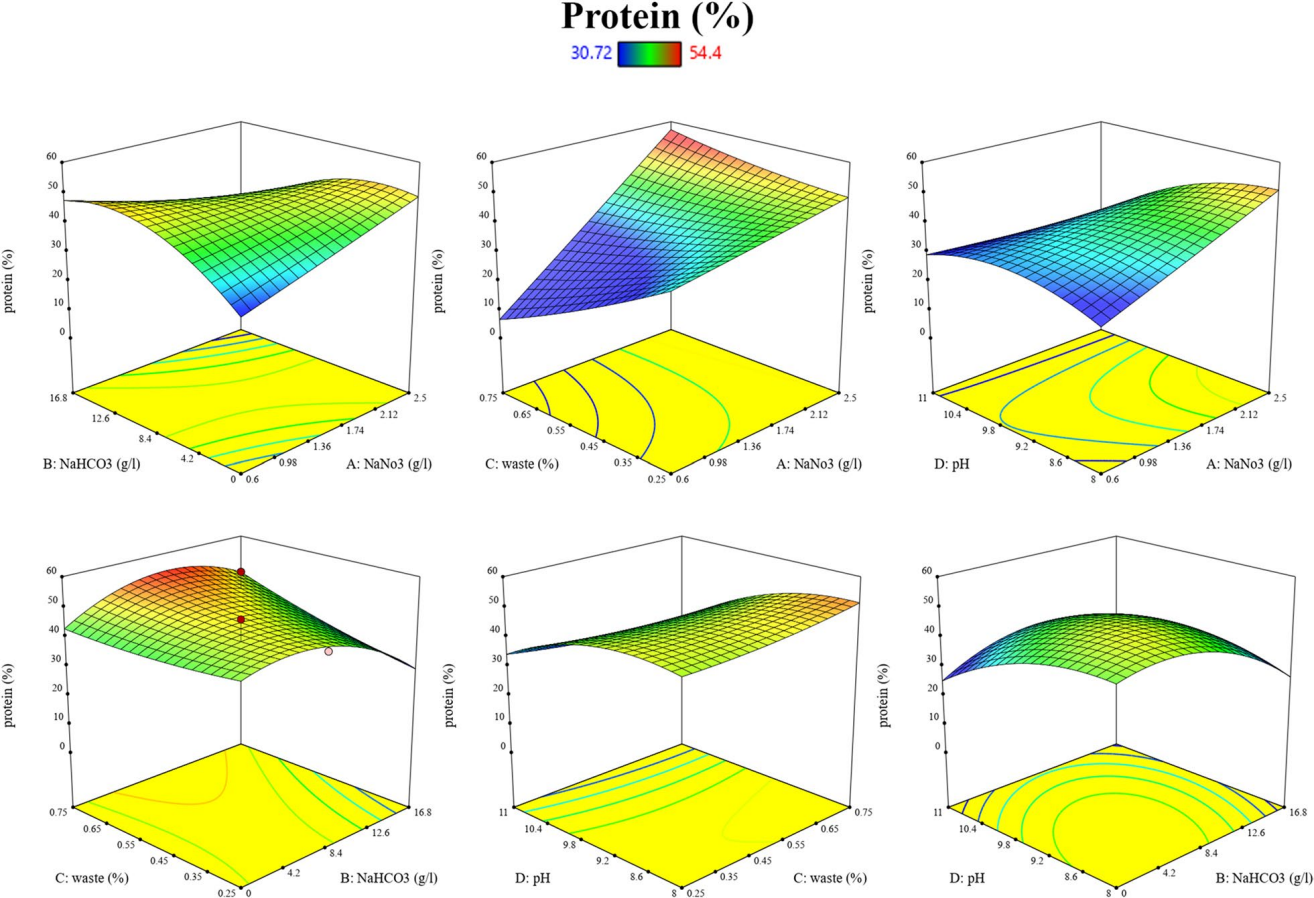
Three-dimensional response surface plot showing the interaction between the tested variables for optimum protein content production

The interactive effect of variables on protein content was revealed by response surface plots (Fig. 16). It can be noted that an increase in protein content was obtained at NaNO_3_ 2.5 g/L and NaHCO_3_ from 2.1 to 6.3 g/L. The interaction between NaNO_3_ and waste showed that the optimum NaNO_3_ and waste range was 2.12–2.5 g/L and 35–75%, producing 50–60% protein. Moreover, an enhanced protein response was observed at the interaction between NaNO_3_ and pH, with optimum ranges of 2.4–2.5 g/L and 8–8.9, respectively.

This result is in agreement with Colla et al. [49], they stated that the lower sodium nitrate concentrations (0.625 and 1.250 g/L) gave lower values for cellular proteins, while the highest levels of protein being obtained in Zarrouk’s medium containing 1.875 or 2.500 g/L sodium nitrate.

Regarding the interaction between NaHCO_3_ and waste, the optimum NaHCO_3_ and waste ranges for maximum protein production were 4–12.6 g/L and 60–75%, respectively. Additionally, in the interactions between NaHCO_3_ and pH, the optimum NaHCO_3_ and pH ranges were 1.05–8.4 g/L and 8–9.5, respectively. Ultimately, the protein response was enhanced by the interaction between waste and pH, with optimum ranges of 67–75% and pH 8–8.9.

During the optimization process, the statistical tool produced 31 trials with several combinations of all independent variables. The predicated combination of independent variables showing the highest desirability value was selected as the predicted optimization value. The best biomass yield (0.8 g/L) and protein (54.15%) were with the following level of independent variables: NaNO_3_ (2.5 g/L), NaHCO_3_ (0.678 g/L), BFCE (33.08%) and pH = 8.

The model was verified experimentally. The actual results were then compared with the predicted value of the models in Eqs. (1) and (2), and the obtained values are shown in Table 4.

**Table 4:** The actual and predicted values of the growth variables for maximizing each response

Variables				Actual value		Predicated value	
NaNO_3_ (g/L)	NaHCO_3_ (g/L)	BFCE (%)	pH	Biomass (g/L)	Protein (%)	Biomass (g/L)	Protein (%)
2.5	0.678	0.3308	8	0.566	52.58	0.8	54.15

#### Amino acids profile of *S. platensis*

The protein quality of *S. platensis* depends mainly on its amino acid content, especially essential amino acids [50]. The result concerning the amino acids profile of *S. platensis* cultivated in SZM and optimized medium are shown in Table 5. The result of essential amino acid in *S. platensis* protein exhibited; was mainly leucine (8.36%) followed by valine (5.71%), whilst the lowest percentage was noted for histidine (1.62%) in the optimized medium. Likewise, leucine (8.16%) and valine (5.4%) were the major essential amino acids in SZM. Fortunately, the total essential amino acid was higher in the optimized medium (38.73%) than in SZM (36.98%). The results for non-essential amino acids in *S. platensis* protein indicated that glutamic acid was the major amino acid up to 14.65% and 14.26% in optimized and SZM, respectively. In contrast, cysteine was the lowest content (0.31%). It is evident from the results that TEAA in *S. platensis* protein from the optimized medium using BFCE was a promising source of leucine, valine, isoleucine, lysine, glutamic acid, aspartic acid, alanine and arginine compared to the SZM. These levels and profile of amino acids in *Spirulina* are similar to that reported by Bashir et al. [51]

**Table 5:** The essential and non-essential amino acid of *S. platensis* protein in SZM and optimized medium

Essential amino acid	SZM (%)	Optimized (%)	Non-essential amino acid	SZM (%)	Optimized (%)
Threonine (THR)	4.18	4.99	Glutamic (GLU)	14.26	14.65
Valine (VAL)	5.4	5.71	Aspartic (ASP)	9.11	9.5
Isoleucine (ISO)	5.21	5.4	Glycine (GLY)	4.6	4.96
Leucine (LEU)	8.16	8.36	Alanine (ALA)	6.49	6.96
Phenylalanine (PHE)	4.65	4.93	Tyrosine (TYR)	1.67	2.67
Histidine (HIS)	1.78	1.62	Arginine (ARG)	5.93	6.16
Lysine (LYS)	4.48	4.57	Proline (PRO)	3.54	3.82
Methionine (MET)	3.12	3.15	Cystine (CYS)	0.31	1.67
TEAA	36.98	38.73	Serine (SER)	3.2	3.87

*TEAA* total essential amino acids, *SZM* standard Zarrouk medium

## Conclusion

Microalga *S. platensis* can adapt to high concentrations of BFCE up to 75% substitution of SZM. Furthermore, the biomass and protein content similar to that of SZM. In contrast, direct cultivation on higher concentrations of BFCE led to low protein content and biomass compared to the SZM. The CCD predicated the optimum variables of NaNO_3_ (2.5 g/L), NaHCO_3_ (0.6 g/L), BFCE (33%), and pH = 8 to maximize the biomass yield and protein content. The resulting biomass yield was 0.56 g/L, and protein content was up to 52.5%. Also, protein quality was determined by its total essential amino acids content, which was 38.73% in the optimized medium and 36.98% in SZM. Economically, the newly optimized medium significantly saves approximately 96% NaHCO_3_ compared to SZM without affecting the protein content and biomass production, and these results indicating that BFCE could be used as a promising feedstock for the cost-effective large-scale cultivation of *Spirulina* as a microbial cell factory for protein*.*

## Materials and methods

### Collection, preparation, and chemical composition of BFC aqueous extract

The BFC was collected from Dakahlia Sugar Factory, Dakahlia province, Egypt. A hundred grams of solid waste was transferred into a pre-weighed 2 L screw cap Schott Duran^®^ bottle (w_1_) containing 1 L distilled water, autoclaved at 121 °C, 1.5 atm for 20 min. After autoclaving, the bottle was left to settle down, and then the clear beet filter cake extract (BFCE) was separated under aseptic conditions. Complete characterization of BFCE ingredients, including nitrate, ammonium, and phosphorus, was carried out. To confirm the toxic pollutants' absence, the concentration of the minerals was detected using inductively coupled plasma mass spectrometry (ICP-MS) before *S. platensis* inoculation.

### Cultivation of *S. platensis* on different concentrations of BFCE

The BFCE-supplemented cultures were prepared by adding different volumes of beet filter cake extract (BFCE) to the standard Zarrouk’s medium (SZM) [52]**,** while SZM only served as the control treatment. Two strategies were applied in this study as following.

#### Batch culture experiment

*S. platensis* was cultivated in SZM supplemented with BFCE. The constituents of SZM were eliminated stepwise and substituted with increasing percentage volume of BFCE (SZM, 25%, 50%, 75% and 100% BFCE). The experiment was conducted in a replicate for each treatment. All treatments proceeded with a total volume of 400 mL in a 500 mL Erlenmeyer flask, then inoculated with 10% (v/v) exponentially growing culture (O.D = 0.8 at 680 nm). At the same time, the incubation temperature was 26 ± 2 °C, 50 µmol/s/m^2^ illuminations, 16:8 h light: dark cycle, and continuous bubbling with sterile air flow for seven days.

#### Incremental waste acclimatization experiment

In this design, the culture was subjected to a gradual increment in the BFCE concentration for each run, while the control was only SZM. In the first run, *S. platensis* inoculum was inoculated in the SZM and the 25% BFCE. Then after one week, each flask in the experiment was equally divided into two fractions (200 mL each) and centrifuged at 6000 rpm for 10 min. The first fraction’s pellet was used for further analysis after lyophilization. In contrast, the other fraction’s pellet was used as an inoculum in the next run at an increment BFCE medium ratio equal to 25% BFCE higher than the current concentration. At the same time, the control flasks followed the same steps but with the same SZM for the next run. As a result, a total of four runs with BFCE concentrations of 25, 50, 75 and 100% for 28 days was the whole duration of the experiment, while the control for each run was still the same SZM constituents.

### Analytical methods

#### Growth parameters determination

For the photosynthetic performance assessment, chlorophyll fluorescence was measured daily in dark-adapted cultures of *S. platensis* grown in SZM and BFCE using a pulse amplitude modulation fluorometer (PAM) (AquaPen AP 110-C). The maximum photochemical efficiency of photosystem II (Fv/Fm) was measured [53, 54]. The culture optical density was also measured daily using a spectrophotometer at a wavelength of 680 nm [55]. For dry weight estimation, the culture of *S. platensis* was harvested by centrifugation at 6000 rpm for 10 min and washed with distilled water to remove any salt remains. The pellet of the *S. platensis* was dried in a lyophilizer for 48 h at − 50 °C and weighed [56].

#### Estimation of chlorophyll-*a*, *b* and carotenoids contents

The pigment fraction (chlorophyll-*a*, *b* and carotenoids) was extracted by a known volume of 80% acetone and then measured spectrophotometrically at wavelengths 664, 647, 630 and 452 nm, respectively. The pigment concentrations were calculated by Jeffrey St, et al. [57] equations.

#### Biochemical characteristic determination

Protein content was determined according to Lowry’s method [58]. The protein estimation was carried out by cell lysis using bovine serum albumin (BSA) as a standard. Total carbohydrates were determined according to the phenol sulfuric acid method described by Dubois et al. [59], and the concentration of carbohydrates was calculated from the standard curve of glucose. Lipid concentration was estimated by the sulfo-phospho-vanillin method described by Byreddy et al. [60], and the lipid content was calculated using a standard curve of cholesterol.

### Experimental design for optimization of the protein content and growth yield in the *S. platensis* using central composite design

Central composite design (CCD) is the most popular RSM design and was applied to determine the combined influence of two or more variables used in the experiment. Based on the results of the previous experiments, four independent variables, namely (nitrate concentration (g/L), sodium bicarbonate concentration (g/L), BFCE concentration (%), and pH value) and two dependent variables, namely: biomass yield (g/L) and protein content (%), were chosen for designing the experiment by CCD in Minitab^©^ software with five levels of variables for selected factors (− 2, − 1, 0, + 1, + 2) as shown in Table 6.

**Table 6:** Code limit for variables used in the experiment design

Factor	Unit	Symbol	Levels				
			− 2	− 1	0	+ 1	+ 2
NaNo_3_	(g/L)	X1	0.6	1.075	1.55	2.025	2.5
NaHCO_3_	(g/L)	X2	0	4.2	8.4	12.6	16.8
BFCE	(%)	X3	0.25	0.375	0.5	0.625	0.75
pH	–	X4	8	8.75	9.5	10.25	11

CCD design generated 31 runs with seven replicates of central points, in addition to the control flask containing only SZM. Each flask was inoculated with 40 mL of *S. platensis* inoculum and incubated under optimum culturing conditions. The photosynthetic activity and optical density at OD = 680 nm were measured daily, and at the end of the experiment the biomass yield was determined by dry weight and protein content by Lowry’s method [58].

The predicted optimum results were calculated according to the following equation [61].

3 $${\text{Y}} = \beta 0 + \sum \beta {\text{ i Xi}} + \sum \beta {\text{ ii Xii}} + \sum \beta {\text{ij Xij}}$$

where Y is the response, βi is the regression coefficient for each factor, βii is the regression coefficient for square effects, and βij is the regression coefficient for interaction.

The data obtained were analyzed using analysis of variance (ANOVA) using Design Expert 8 software. Based on the analysis of results, validation experiment for CCD was carried out.

#### Amino acid profile

The amino acids content of *S. platensis* grown in SZM and optimized medium was determined at Regional Center for Food and Feed (RCFF), Egypt, by using AOAC Method No. 994.12E [62] using high performance amino acid analyzer (Biochrom 30 plus series of Amino Acid Analyzer System (Biochrom, Cambridge, UK).

### Statistical analysis

The mean, and standard error were calculated from the triplicate experimentations. The one-way ANOVA was applied to identify the significant differences between treatments using Duncan’s test at a significant level p ≤ 0.05. Growth medium optimization was performed through CCD method using Minitab^©^ software. CCD consisting of variables and responses were analyzed statistically and subjected to analysis of variance (ANOVA) to evaluate “the fit of the model” and the *R*^*2*^ (coefficient determination) value was calculated by the regression equations.

## Supplementary Information

**Additional file 1: Table S1**. Growth parameters (optical density and photosynthetic activity) of *S*. *platensis* cultivated under different concentrations of NaNO_3_, NaHCO_3_, BFCE and pH in Central Composite Design. **Fig. S1**. Growth of *S. platensis* cultivated in the SZM and different BFCE treatments. **Fig. S2**. Growth of *S*. *platensis* cultivated in the SZM and BFCE treatments for incremental waste acclimatization for 28 days. **Fig. S3**. Optimization of growth parameters using Central Composite Design (CCD) for eight days of incubation.

## Data Availability

All data generated and analyzed during this study are included in this manuscript and in its Additional file.

## Abbreviations

ANOVA: Analysis of variance
AOAC: Association of Official Agricultural Chemists
BFC: Beet filter cake
BFCE: Beet filter cake extract
CCD: Central composite design
Fv/Fm: Maximum quantum yield
ICP-MS: Inductively coupled plasma mass spectrometry
PAM: Pulse amplitude modulation
RCFF: Regional Center for Food & Feed
RSM: Response surface methodology
SCP: Single-cell protein
SZM: Standard Zarrouk’s media
TEAA: Total essential amino acids
βi: Regression coefficient for each factor
βii: Regression coefficient for square effects
βij: Regression coefficient for interaction
