# Supplementary material for: Filter cake extract from the beet sugar industry as an economic growth medium for the production of Spirulina platensis as a microbial cell factory for protein
Source: Microb Cell Fact. 2023 Jul 24;22:136. doi: 10.1186/s12934-023-02146-7 (PMC10367415; doi:10.1186/s12934-023-02146-7)
Supplement: Supplementary file 1 — Additional file 1: Table S1. Growth parameters (optical density and photosynthetic activity) of S. platensis cultivated under different concentrations of NaNO3, NaHCO3, BFCE and pH in Central Composite Design. Fig. S1. Growth of S. platensis cultivated in the SZM and different BFCE treatments. Fig. S2. Growth of S. platensis cultivated in the SZM and BFCE treatments for incremental waste acclimatization for 28 days. Fig. S3. Optimization of growth parameters using Central Composite Design (CCD) for eight days of incubation. [file 12934_2023_2146_MOESM1_ESM.docx]

Additional files for microbial cell factories

**Filter cake extract from the beet sugar industry as an economic growth medium for the production of *Spirulina platensis* as a microbial cell factory for protein**

**Sara Saad^1^, Mervat Hosny Hussien^1^, Ghada Samir Abou-ElWafa^1^, Heshmat Soliman Aldesuquy^1^, and Eladl Eltanahy^1^**

^1^ Botany Department, Faculty of Science, Mansoura University, Mansoura 35516, Egypt.

*** Corresponding author:** Eladl Eltanahy, [eladl@mans.edu.eg](mailto:eladl@mans.edu.eg)

Table S1. Growth parameters (optical density and photosynthetic activity) of S platensis cultivated under different concentrations of NaNO_3_, NaHCO_3_, BFCE and PH in Central Composite Design.

|  | | Incubation time (days) | | | | | | | |
| --- | --- | --- | --- | --- | --- | --- | --- | --- | --- |
| run | index | 1 | 2 | 3 | 4 | 5 | 6 | 7 | 8 |
| 1 | OD680 | 0.319 | 0.28 | 0.276 | 0.407 | 0.47 | 0.657 | 0.788 | 0.871 |
|  | Fv/Fm | 0.555 | 0.551 | 0.543 | 0.499 | 0.487 | 0.502 | 0.493 | 0.469 |
| 2 | OD680 | 0.287 | 0.316 | 0.371 | 0.456 | 0.55 | 0.843 | 0.919 | 1.04 |
|  | Fv/Fm | 0.543 | 0.555 | 0.534 | 0.514 | 0.524 | 0.493 | 0.467 | 0.447 |
| 3 | OD680 | 0.298 | 0.306 | 0.388 | 0.414 | 0.57 | 0.685 | 0.797 | 0.862 |
|  | Fv/Fm | 0.511 | 0.539 | 0.541 | 0.527 | 0.508 | 0.501 | 0.475 | 0.46 |
| 4 | OD680 | 0.293 | 0.353 | 0.366 | 0.513 | 0.596 | 0.804 | 1.002 | 1.124 |
|  | Fv/Fm | 0.538 | 0.553 | 0.563 | 0.545 | 0.528 | 0.479 | 0.46 | 0.454 |
| 5 | OD680 | 0.338 | 0.48 | 0.394 | 0.535 | 0.591 | 0.856 | 0.924 | 1.003 |
|  | Fv/Fm | 0.452 | 0.476 | 0.511 | 0.503 | 0.492 | 0.478 | 0.454 | 0.444 |
| 6 | OD680 | 0.351 | 0.401 | 0.412 | 0.565 | 0.565 | 0.788 | 0.977 | 0.986 |
|  | Fv/Fm | 0.456 | 0.495 | 0.522 | 0.505 | 0.495 | 0.477 | 0.451 | 0.436 |
| 7 | OD680 | 0.294 | 0.385 | 0.393 | 0.548 | 0.641 | 0.779 | 0.906 | 1.043 |
|  | Fv/Fm | 0.487 | 0.545 | 0.537 | 0.53 | 0.509 | 0.498 | 0.478 | 0.461 |
| 8 | OD680 | 0.288 | 0.366 | 0.402 | 0.544 | 0.639 | 0.794 | 0.945 | 1.04 |
|  | Fv/Fm | 0.451 | 0.47 | 0.511 | 0.509 | 0.517 | 0.473 | 0.46 | 0.444 |
| 9 | OD680 | 0.36 | 0.44 | 0.318 | 0.642 | 0.59 | 0.616 | 0.773 | 0.8 |
|  | Fv/Fm | 0.418 | 0.457 | 0.481 | 0.499 | 0.508 | 0.519 | 0.52 | 0.525 |
| 10 | OD680 | 0.332 | 0.424 | 0.369 | 0.566 | 0.664 | 0.771 | 0.812 | 0.95 |
|  | Fv/Fm | 0.504 | 0.545 | 0.535 | 0.533 | 0.536 | 0.521 | 0.521 | 0.5 |
| 11 | OD680 | 0.31 | 0.389 | 0.555 | 0.582 | 0.697 | 0.814 | 0.978 | 0.987 |
|  | Fv/Fm | 0.414 | 0.448 | 0.515 | 0.541 | 0.529 | 0.492 | 0.484 | 0.488 |
| 12 | OD680 | 0.298 | 0.45 | 0.455 | 0.55 | 0.654 | 0.791 | 0.918 | 0.973 |
|  | Fv/Fm | 0.445 | 0.49 | 0.53 | 0.554 | 0.516 | 0.481 | 0.469 | 0.455 |
| 13 | OD680 | 0.394 | 0.5 | 0.645 | 0.51 | 0.773 | 0.883 | 1.039 | 1.168 |
|  | Fv/Fm | 0.474 | 0.517 | 0.52 | 0.519 | 0.499 | 0.502 | 0.493 | 0.517 |
| 14 | OD680 | 0.407 | 0.527 | 0.608 | 0.542 | 0.783 | 0.94 | 1.008 | 1.058 |
|  | Fv/Fm | 0.461 | 0.512 | 0.522 | 0.526 | 0.493 | 0.531 | 0.488 | 0.477 |
| 15 | OD680 | 0.337 | 0.366 | 0.54 | 0.62 | 0.8 | 0.973 | 1.056 | 0.706 |
|  | Fv/Fm | 0.454 | 0.495 | 0.515 | 0.525 | 0.499 | 0.461 | 0.467 | 0.452 |
| 16 | OD680 | 0.295 | 0.385 | 0.46 | 0.491 | 0.693 | 0.8 | 0.935 | 1.073 |
|  | Fv/Fm | 0.506 | 0.487 | 0.532 | 0.549 | 0.524 | 0.475 | 0.473 | 0.461 |
| 17 | OD680 | 0.333 | 0.39 | 0.489 | 0.442 | 0.731 | 0.938 | 1.062 | 1.103 |
|  | Fv/Fm | 0.465 | 0.549 | 0.513 | 0.505 | 0.498 | 0.454 | 0.451 | 0.424 |
| 18 | OD680 | 0.291 | 0.366 | 0.432 | 0.451 | 0.713 | 0.873 | 0.988 | 1.072 |
|  | Fv/Fm | 0.477 | 0.511 | 0.523 | 0.524 | 0.516 | 0.492 | 0.475 | 0.461 |
| 19 | OD680 | 0.317 | 0.36 | 0.441 | 0.473 | 0.633 | 0.847 | 0.916 | 0.73 |
|  | Fv/Fm | 0.466 | 0.542 | 0.577 | 0.559 | 0.537 | 0.497 | 0.498 | 0.481 |
| 20 | OD680 | 0.294 | 0.383 | 0.476 | 0.602 | 0.705 | 0.87 | 0.957 | 1.13 |
|  | Fv/Fm | 0.471 | 0.495 | 0.532 | 0.535 | 0.512 | 0.477 | 0.456 | 0.442 |
| 21 | OD680 | 0.338 | 0.344 | 0.479 | 0.573 | 0.737 | 0.902 | 1.041 | 1.149 |
|  | Fv/Fm | 0.444 | 0.494 | 0.508 | 0.511 | 0.497 | 0.467 | 0.462 | 0.448 |
| 22 | OD680 | 0.321 | 0.349 | 0.519 | 0.593 | 0.79 | 0.927 | 1.006 | 1.202 |
|  | Fv/Fm | 0.551 | 0.532 | 0.524 | 0.527 | 0.502 | 0.43 | 0.431 | 0.417 |
| 23 | OD680 | 0.26 | 0.377 | 0.615 | 0.619 | 0.798 | 0.838 | 0.999 | 1.105 |
|  | Fv/Fm | 0.465 | 0.507 | 0.488 | 0.504 | 0.497 | 0.46 | 0.487 | 0.419 |
| 24 | OD680 | 0.353 | 0.429 | 0.553 | 0.623 | 0.69 | 0.87 | 0.988 | 1.04 |
|  | Fv/Fm | 0.476 | 0.533 | 0.541 | 0.544 | 0.533 | 0.488 | 0.468 | 0.479 |
| 25 | OD680 | 0.321 | 0.293 | 0.375 | 0.557 | 0.685 | 0.822 | 0.96 | 1.091 |
|  | Fv/Fm | 0.472 | 0.503 | 0.511 | 0.508 | 0.504 | 0.484 | 0.456 | 0.443 |
| 26 | OD680 | 0.329 | 0.383 | 0.467 | 0.58 | 0.659 | 0.883 | 0.974 | 1.126 |
|  | Fv/Fm | 0.504 | 0.515 | 0.514 | 0.512 | 0.502 | 0.457 | 0.479 | 0.441 |
| 27 | OD680 | 0.309 | 0.276 | 0.484 | 0.615 | 0.69 | 0.762 | 0.876 | 1.141 |
|  | Fv/Fm | 0.557 | 0.566 | 0.544 | 0.521 | 0.506 | 0.485 | 0.464 | 0.452 |
| 28 | OD680 | 0.32 | 0.285 | 0.463 | 0.526 | 0.67 | 0.861 | 0.906 | 1.102 |
|  | Fv/Fm | 0.537 | 0.545 | 0.53 | 0.524 | 0.509 | 0.465 | 0.466 | 0.445 |
| 29 | OD680 | 0.326 | 0.336 | 0.408 | 0.698 | 0.67 | 0.842 | 0.956 | 1.125 |
|  | Fv/Fm | 0.556 | 0.562 | 0.526 | 0.533 | 0.5 | 0.465 | 0.453 | 0.454 |
| 30 | OD680 | 0.33 | 0.387 | 0.53 | 0.687 | 0.81 | 0.907 | 0.998 | 1.13 |
|  | Fv/Fm | 0.479 | 0.537 | 0.515 | 0.532 | 0.495 | 0.452 | 0.482 | 0.445 |
| 31 | OD680 | 0.29 | 0.403 | 0.554 | 0.432 | 0.632 | 0.74 | 0.765 | 0.954 |
|  | Fv/Fm | 0.525 | 0.528 | 0.506 | 0.535 | 0.508 | 0.483 | 0.469 | 0.456 |
| Control | OD680 | 0.220 | 0.306 | 0.500 | 0.688 | 0.729 | 0.802 | 0.917 | 1.320 |
|  | Fv/Fm | 0.433 | 0.46 | 0.525 | 0.474 | 0.488 | 0.496 | 0.474 | 0.487 |


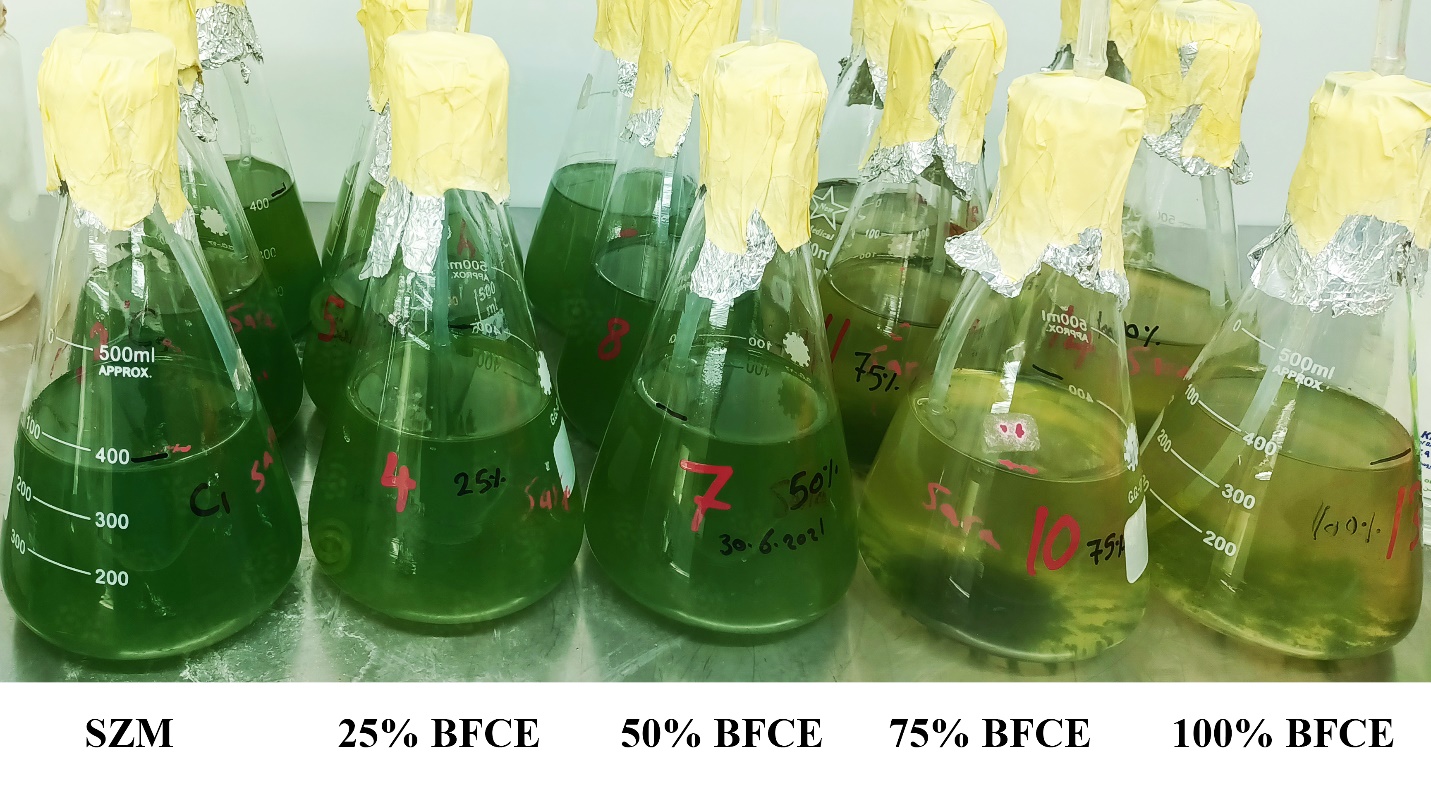


Fig. S1. Growth of S. platensis cultivated in the SZM and different BFCE treatments.


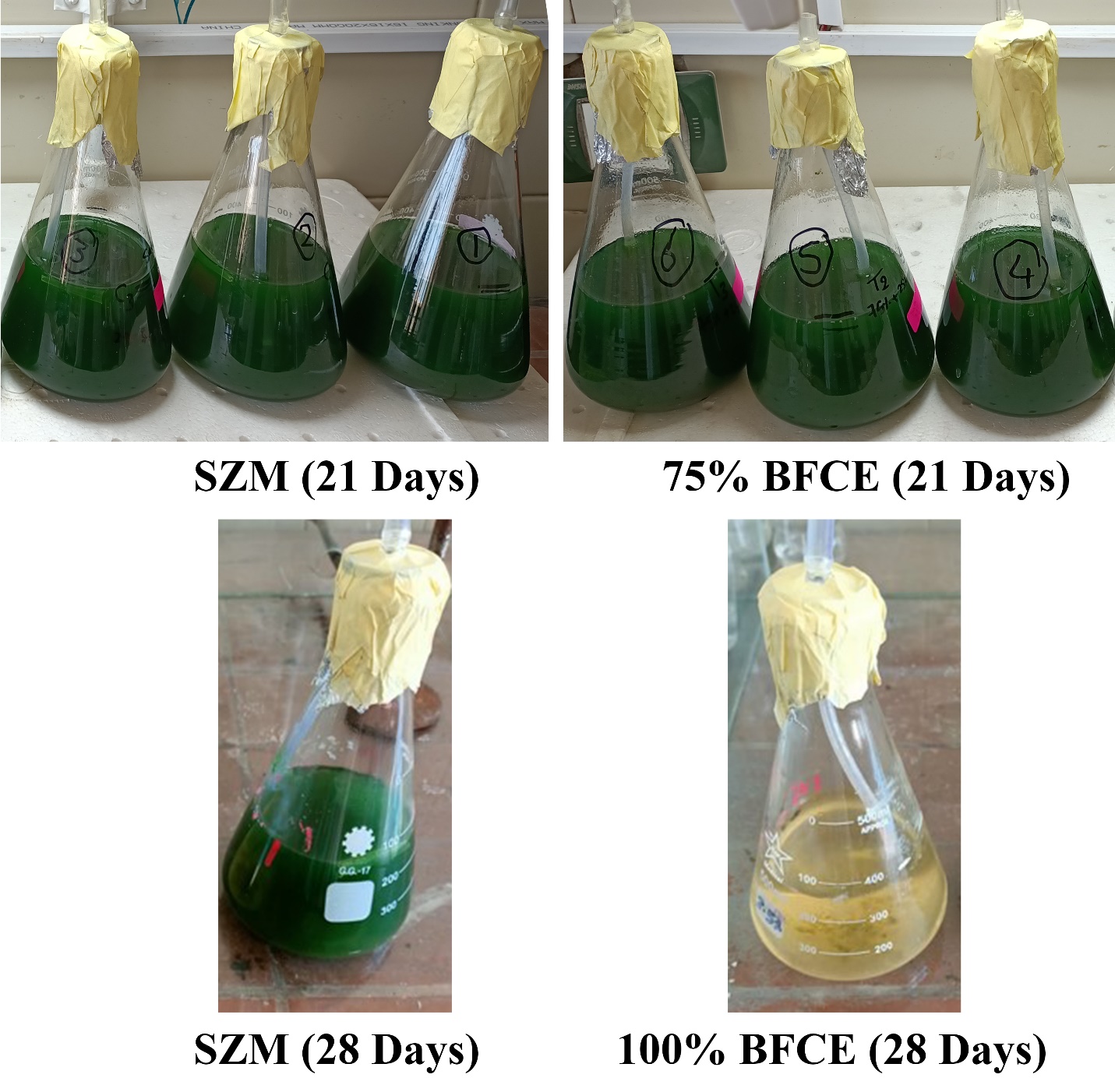


Fig. S2. Growth of S. platensis cultivated in the SZM and BFCE treatments for incremental waste acclimatization for 28 days.


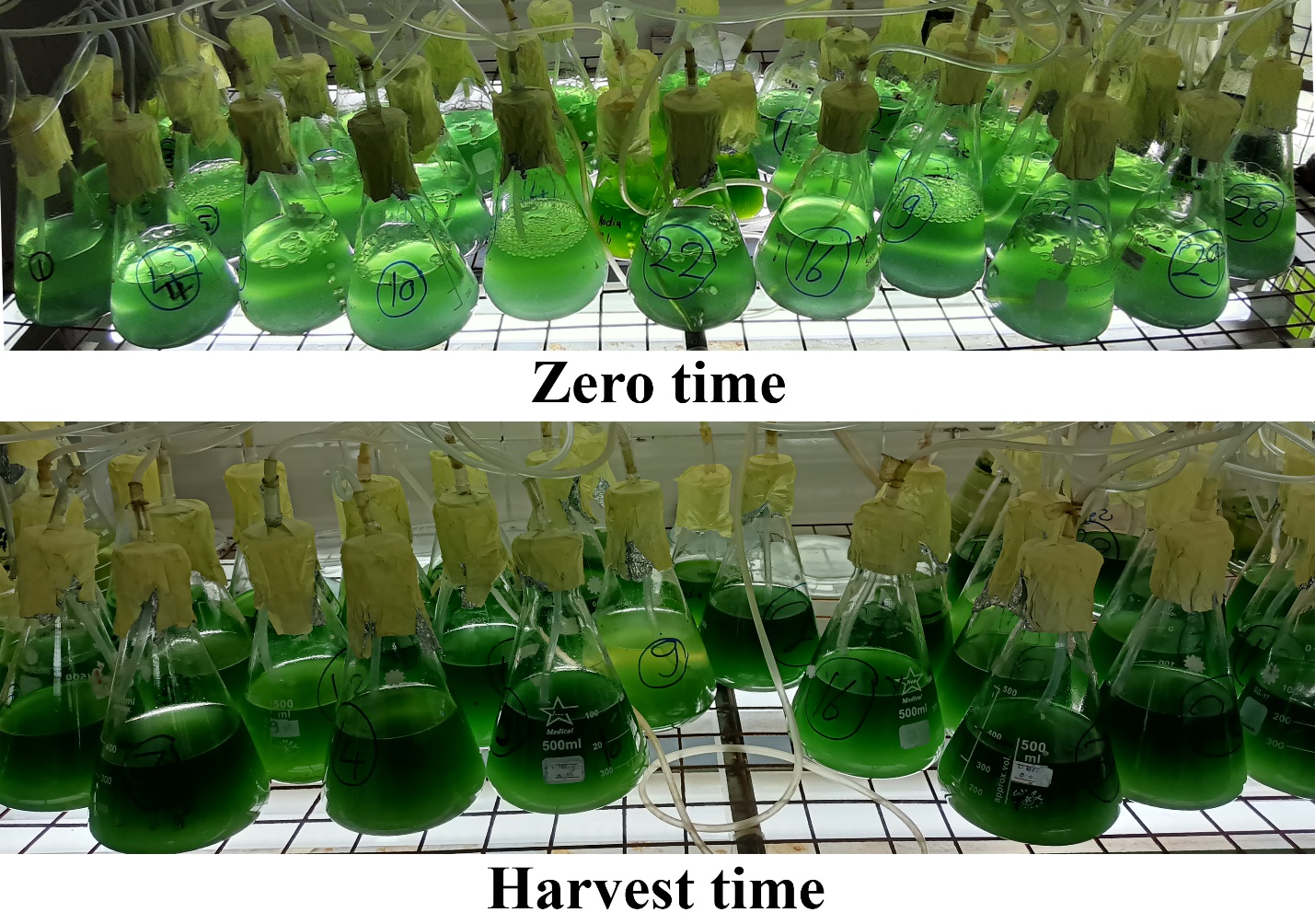


Fig. S3. Optimization of growth parameters using Central Composite Design (CCD) for eight days of incubation.
